# Serotonergic Chemosensory Neurons Modify the *C. elegans* Immune Response by Regulating G-Protein Signaling in Epithelial Cells

**DOI:** 10.1371/journal.ppat.1003787

**Published:** 2013-12-12

**Authors:** Alexandra Anderson, Henry Laurenson-Schafer, Frederick A. Partridge, Jonathan Hodgkin, Rachel McMullan

**Affiliations:** 1 Department of Life Sciences, Imperial College London, South Kensington Campus, London, United Kingdom; 2 Department of Biochemistry, University of Oxford, Oxford, United Kingdom; Massachusetts General Hospital, Harvard Medical School, United States of America

## Abstract

The nervous and immune systems influence each other, allowing animals to rapidly protect themselves from changes in their internal and external environment. However, the complex nature of these systems in mammals makes it difficult to determine how neuronal signaling influences the immune response. Here we show that serotonin, synthesized in *Caenorhabditis elegans* chemosensory neurons, modulates the immune response. Serotonin released from these cells acts, directly or indirectly, to regulate G-protein signaling in epithelial cells. Signaling in these cells is required for the immune response to infection by the natural pathogen *Microbacterium nematophilum*. Here we show that serotonin signaling suppresses the innate immune response and limits the rate of pathogen clearance. We show that *C. elegans* uses classical neurotransmitters to alter the immune response. Serotonin released from sensory neurons may function to modify the immune system in response to changes in the animal's external environment such as the availability, or quality, of food.

## Author Summary

The nervous and immune systems respond quickly and precisely to changes in the environment. Communication between these systems may underlie neurological disorders such as depression, and explain why environmental factors, such as psychological stress, increase infection susceptibility. What are the molecular mechanisms that link the nervous and immune systems? *C. elegans* has a well described nervous system and can be infected by several pathogens, making it an appropriate model organism with which to address this question. We show that synthesis of the neurotransmitter serotonin, in sensory neurons exposed to the environment, alters susceptibility to infection with *Microbacterium nematophilum*. Unlike infection with *Pseudomonas aeruginosa*, where serotonin signaling is required for behavioral avoidance, here serotonin signaling suppresses the immune response by regulating a G-protein signaling pathway in epithelial cells. Thus, we show that altering levels of serotonin can trigger different outcomes depending on the environment. We identify a single neuron capable of modifying the immune response and demonstrate that *C. elegans* uses classical neurotransmitters to modify its immunity. Our work demonstrates that *C. elegans* can be used to study reciprocal cross talk between neurotransmitters and immune responses that may be important for the pathology of disorders including depression.

## Introduction

The nervous and immune systems respond quickly and precisely to the presence of pathogenic microbes in an animal's environment. Whilst the immune system activates cellular defenses to recognize and eliminate pathogens, changes in neuronal signaling alter animal behavior to avoid these microbes. Data from mammalian models suggests that bidirectional communication between these two systems can modify responses to infection [1], and this relationship may explain why psychological stress increases susceptibility to infections [2]. Because of the complicated nature of the mammalian brain and immune system the molecular mechanisms that underlie neuronal regulation of the immune response remain unclear.

The free-living soil nematode *Caenorhabditis elegans* utilizes conserved signaling pathways to trigger behavioral and innate immune responses to infection by several natural and clinically-relevant pathogens provided as a food source [3]. This, together with its simple and well-described nervous system, has resulted in several studies identifying the neuronal signals that influence *C. elegans* behavioral and immune responses to infection [4]–[9]. In the presence of pathogens such as *Serratia marcescens* [8], *Microbacterium nematophilum* [9] and *Pseudomonas aeruginosa* [5], [6] *C. elegans* uses chemosensory neurons to recognize the pathogen triggering changes in neuronal signaling that cause it to alter its behavior and avoid these potential harmful bacteria. Interestingly, neuronal signals can also directly modify *C. elegans* immune responses. Release of the insulin like neuropeptide INS-7 from neuronal dense core vesicles suppresses the *C. elegans* intestinal immune response triggered by infection with *P. aeruginosa* PA14 [4] and this pathway is utilized by the pathogen to suppress host immune defenses [10]. The neuronal cytokine DBL-1(TGFβ) promotes expression of the caenacin family antimicrobial peptide *cnc-2* in the epidermis during infection with the fungal pathogen *Drechmeria conispora* [7]. Several studies have implicated mammalian neuropeptides and peptide hormones in neuronal regulation of immunity (reviewed in [11]) suggesting that these relationships are conserved, and studies in *C. elegans* have identified neurons as important modifiers of the immune response. However, although an octopamine receptor has been shown to regulate the *C. elegans* immune response [12], the function of neurotransmitters in *C. elegans* immunity remains unexplored.

In mammals a number of neurotransmitters act on the immune system to modify its function [13]. One of these is the classical monoamine neurotransmitter serotonin (5-hydroxytryptamine) [14], [15]. Dysregulation of mammalian serotonin signaling is associated with mood disorders including depression, and depressed patients show decreased natural killer (NK) cell activity [16]. These cells are important components of the innate immune system linking serotonin signaling to immune regulation. Furthermore NK cell activity can be enhanced by treatment with anti-depressants, such as Prozac, that act as selective serotonin reuptake inhibitors (SSRI's) [17]. NK cells are not the only immune cells affected by serotonin. Several other cells of the immune system express serotonin receptors including dendritic cells [18], macrophages [19] and mast cells [20]. Serotonin affects both innate and adaptive immunity enhancing the proliferation of B [21] and NK cells [22], promoting stimulation of T cells by macrophages [23] and acting as a chemotactic agent for mast cells [20] and eosinophils [24]. However there is still much unknown about how serotonin functions in the immune response. Genetic approaches will be key to understanding the role of serotonin in immune function. Mice lacking the serotonin biosynthetic enzyme, tryptophan hydroxylase TPH1, have some defects in their immune function [25] however TPH1 acts redundantly with TPH2 [26] and TPH1 knockouts still retain the ability to synthesis serotonin in serotonergic regions of the brain [26]. *C. elegans* only has one tryptophan hydroxylase ortholog, *tph-1*, and animals carrying the putative null allele *tph-1(mg280)* are deficient for serotonin production [27] allowing the immune function of neuronal serotonin to be studied *in vivo*.

In *C. elegans* serotonin signaling allows animals to respond to changes in their environment by modulating locomotion [28], feeding [29], defecation [30] and egg laying [1], [31], [32] behaviors. Serotonin signals the presence of food causing starved animals to stop moving when they encounter a bacterial lawn. Animals lacking *tph-1* behave as if they are starved, decreasing their feeding and egg laying rates [2], [27]. Interestingly serotonin signaling is also required for *C. elegans* to respond to infection by *P. aeruginosa* PA14 [3], [5], [6]. Animals that lack *tph-1* are more susceptible to PA14 than wild type animals. However serotonin signaling is not required for the *C. elegans* immune response in this context, and changes in susceptibility of *tph-1* mutant animals are due exclusively to behavioral pathogen avoidance [4]–[9]. Exposure to PA14 increases serotonin levels in chemosensory neurons and promotes aversive learning so that animals that have been previously exposed to PA14 alter their olfactory preferences to avoid these toxic bacteria [6], [8].

Using the natural *C. elegans* pathogen *Microbacterium nematophilum* we have identified a role of serotonin signaling in suppressing the immune response. Wild type *C. elegans* tends to avoid bacterial lawns contaminated with *M. nematophilum* [9] and following infection an immune response is triggered that includes swelling around the rectal opening and upregulation of host defense genes [5], [6], [33], [34]. Unlike during *P. aeruginosa* infection, serotonin signaling was not require for avoidance of *M. nematophilum* but instead suppressed the immune response by activating signaling via the G-protein GOA-1(Gαo) in rectal epithelial cells. This suppression required serotonin synthesis in the chemosensory neuron, ADF, which contacts the animal's environment via ciliated sensory endings, and the serotonin receptors SER-1 and SER-7. Our data demonstrates that *C. elegans* uses the classical neurotransmitter serotonin to modify its immune response. These signals may function to modify the immune system in response to changes in the animal's external environment, such as the availability of food.

## Results

### Exogenous serotonin inhibits the *C. elegans* immune response to *M. nematophilum* infection

**Figure 1:**
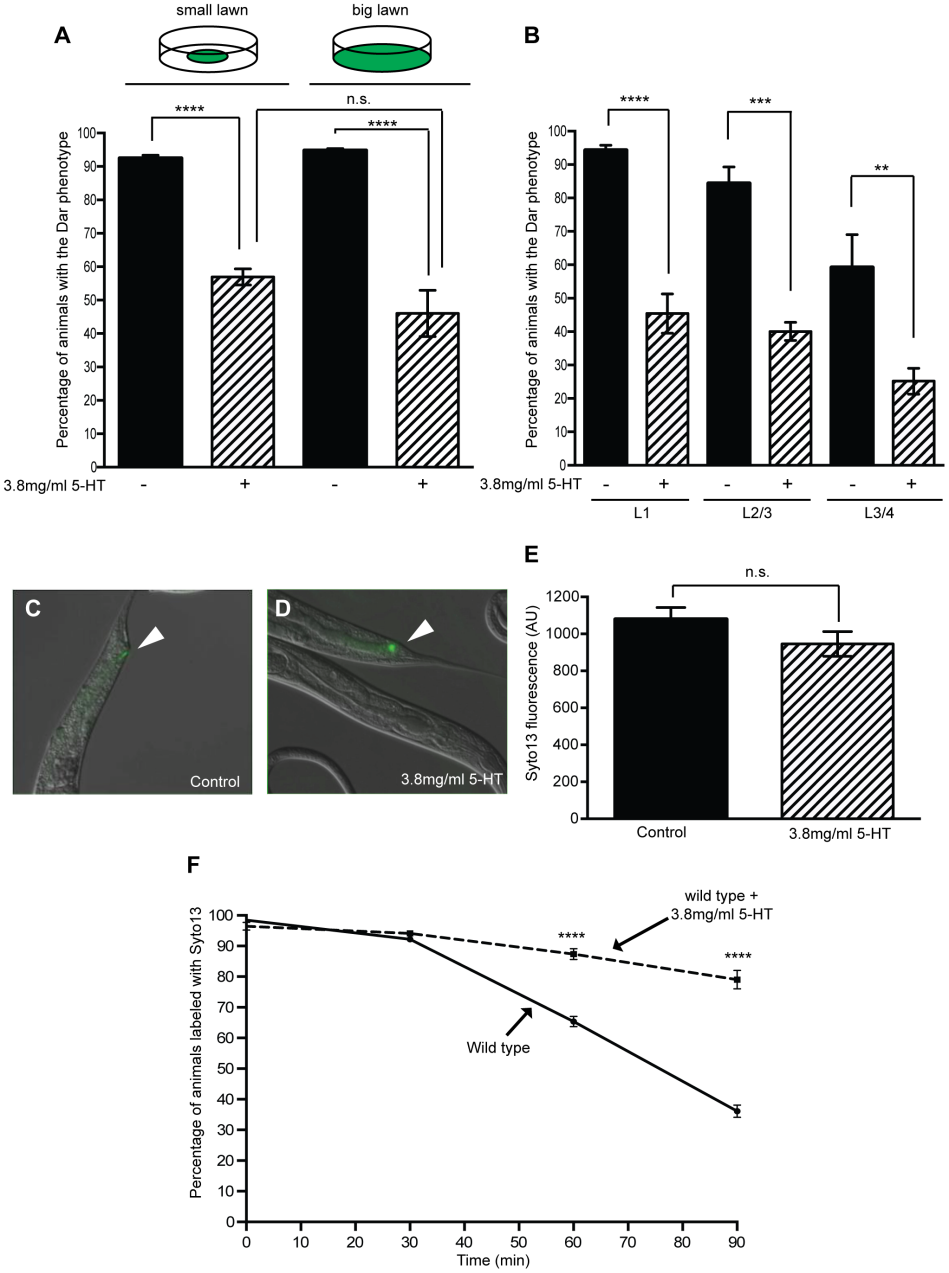
Exogenous serotonin inhibits the Dar phenotype and decreases pathogen clearance rates. Adult wild type animals were exposed to *M. nematophilum* on plates containing exogenous serotonin and the Dar phenotype was scored in their progeny. Treatment with 3.8 mg/ml 5-HT caused a 35% decrease in the number of Dar animals following *M. nematophilum* infection using standard assay conditions (small lawn) (A). A similar decrease was observed when assay conditions were modified so that animals were unable to avoid the pathogen (big lawn) (A). The Dar phenotype was still decreased when wild type animals were infected in the presence of exogenous serotonin during development, at L1 or L2/3 stage, or 10–18 hours prior to adulthood, at L3/4 stage (B). Similar amounts of *M. nematophilum* bacteria, labelled using the nucleic acid stain SYTO13, still attached to the anal opening following serotonin treatment (C, D and E) (the rectal opening is indicated with an arrow head in C and D). SYTO13 labeled *M. nematophilum* was cleared from the anal opening of wild type animals and less than 50% of animals were colonized 90 minutes after transfer to plates without food (F). Treatment of infected animals with 3.8 mg/ml 5-HT significantly decreased the clearance of labeled pathogen from the anal opening (F).

Infection of *C. elegans* with the naturally-occurring pathogen, *Microbacterium nematophilum* triggers an immune response that includes transcription of host defense genes and swelling around the rectal opening, known as the Deformed anal region (Dar) phenotype [4], [33], [34]. To determine whether serotonin was able to modulate the *C. elegans* immune response we exposed adult wild type animals to *M. nematophilum* on plates containing exogenous serotonin and scored the Dar phenotype in their progeny. Treatment with serotonin caused a decrease in the number of Dar animals when compared to untreated controls (Figure 1A and B), however serotonin treatment did not alter the ability of the pathogen to attach to the cuticle. We observed similar levels of SYTO13-labeled *M. nematophilum* adhering to the rectum of serotonin-treated animals that were Dar-defective and control animals (Figure 1C, D and E).

In these experiments animals were exposed to exogenous serotonin throughout development raising the possibility that serotonin treatment alters development to indirectly affect the Dar phenotype. The Dar phenotype requires signaling in the rectal epithelium [35], [36], therefore we first checked that the rectal epithelial marker, LIN-48 (OVO-like transcription factor) [37], was correctly expressed following treatment with exogenous serotonin. Using transgenic animals expressing *lin48p*::FP we did not observe any changes in the expression of this rectal epithelial marker following treatment with exogenous serotonin (Figure S1). To confirm that post-developmental treatment with exogenous serotonin still suppressed the Dar phenotype we also infected animals on plates containing exogenous serotonin at different developmental stages and scored the Dar phenotype when they reached adulthood. Although fewer control animals became Dar when they were infected at the L3/L4 stage (as we have previously observed [10], [36]), we were still able to suppress this Dar phenotype by infecting animals in the presence of exogenous serotonin 10–18 hours prior to adulthood (L3/L4 stage), indicating that exogenous serotonin does not indirectly affect the Dar phenotype by altering development (Figure 1B).

Treatment of *C. elegans* with exogenous serotonin causes dramatic behavioral changes including inhibition of locomotion, stimulation of egg laying and increased pharyngeal pumping [7], [28], [29], [31], [32]. To confirm that defects in these behaviors did not alter the Dar phenotype we infected a number of mutants that phenocopy the effect of exogenous serotonin but do not act in the serotonin signaling pathway. We did not observe any significant differences in the Dar phenotype, when compared to untreated controls, (Table S1) indicating that the effects of serotonin on the immune response are specific and not a secondary consequence of these physiological changes.

Serotonin signaling modulates chemosensory avoidance responses in *C. elegans* [11], [38]. To ensure that the differences we observed reflected a role for serotonin in the immune response, rather than increased behavioral avoidance of the pathogen in the presence of serotonin, we modified our infection assay by spreading *M. nematophilum* to the edges of the plate. In this ‘big-lawn’ assay animals were unable to avoid the pathogen. Animals raised on “big lawns” still displayed a decrease in the Dar phenotype (Figure 1A), indicating that serotonin inhibits the immune response directly rather than modifying *C. elegans* exposure to the pathogen.

### Serotonin synthesis in chemosensory neurons inhibits the Dar phenotype

**Figure 2:**
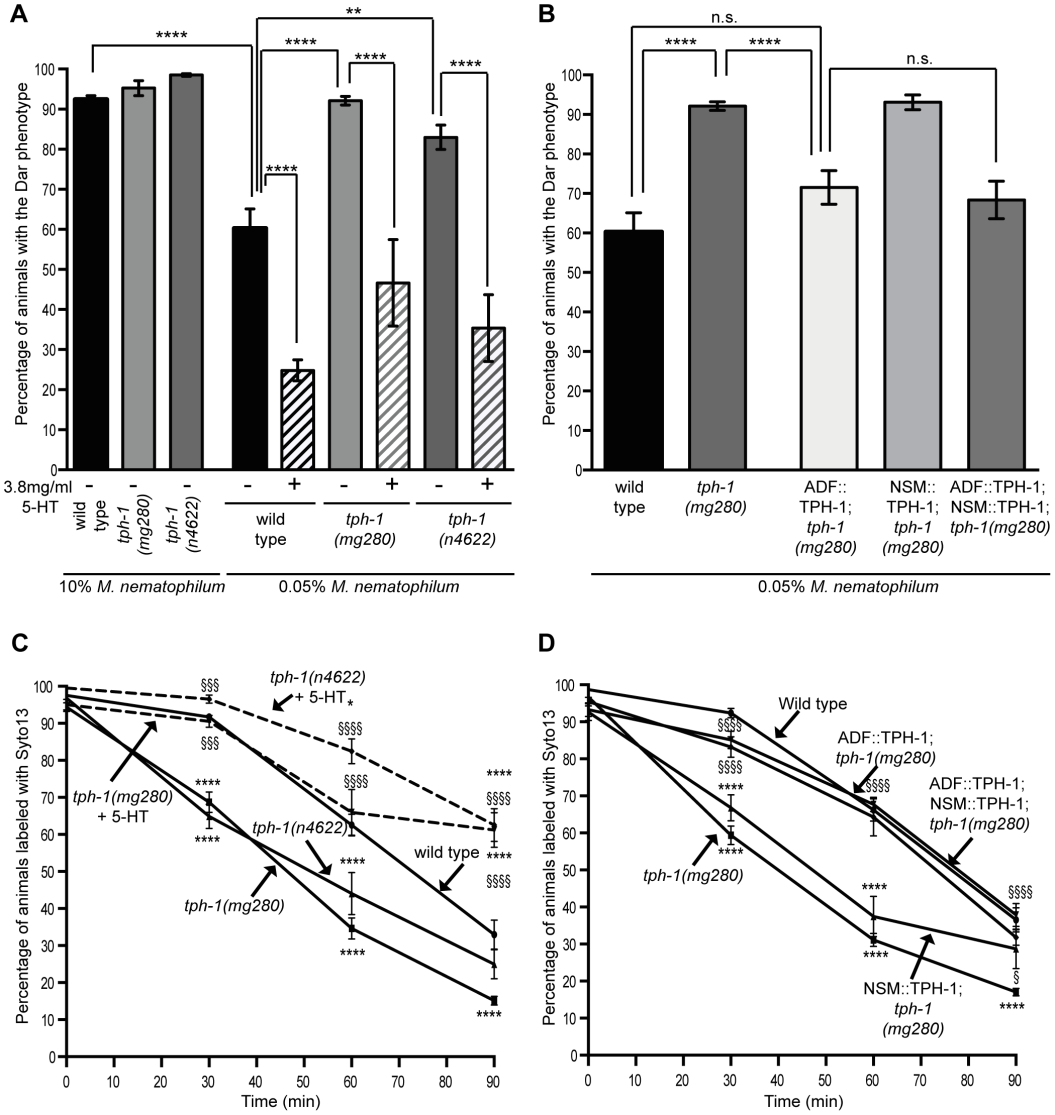
The serotonin biosynthetic enzyme TPH-1 is required in chemosensory neurons to inhibit the Dar phenotype and decrease pathogen clearance rates. Adult *tph-1(mg280)* or *tph-1(n4622)* animals lacking the serotonin biosynthetic enzyme TPH-1 were infected on lawns contaminated with 10% *M. nematophilum*. The percentage of *tph-1(mg280)* and *tph-1(n4622)* progeny with the Dar phenotype was indistinguishable from wild type (A). When animals were infected on lawns contaminated with 0.05% *M. nematophilum* the Dar phenotype was increased from 60.3% in wild type animals to 92.1% in *tph-1(mg280)* and 83.0% in *tph-1(n4622)* (A). This increase could be rescued by treatment with exogenous 5-HT (A) or expression of TPH-1 cDNA in ADF, but not NSM, neurons (B). *tph-1(mg280)* and *tph-1(n4622)* animals cleared SYTO13 labeled pathogen more quickly than wild type animals consistent with a role for TPH-1 in suppressing the immune response (C). This phenotype was rescued by 5-HT treatment (C) or expression of a TPH-1 cDNA in both ADF and NSM neurons or ADF neurons alone but not by expression in NSM alone (D). * indicates significance relative to wild type. § indicates significance relative to untreated mutant control (C) or *tph-1(mg280)* (D) (see materials and methods for details of statistical analysis).

Exogenous serotonin treatment inhibits the immune response, but does endogenous serotonin signaling suppress the wild type immune response? The *C. elegans* tryptophan hydroxlase gene *tph-1* is required for endogenous serotonin biosynthesis and animals carrying the putative null allele *tph-1(mg280)* are deficient for serotonin production [12], [27]. Because increasing serotonin signaling suppressed the immune response we predicted that blocking serotonin signaling, using *tph-1* mutants, would enhance the immune response. However, approximately 90% of wild type animals are able to mount a Dar immune response to contamination of a bacterial lawn with 10% *M. nematophilum* (Figure 1A and 2A), making it difficult to observe treatments that enhance this phenotype. To determine whether loss of serotonin synthesis enhanced the Dar phenotype we modified our infection assay so that bacterial lawns were contaminated with 0.05% *M. nematophilum*. Under these conditions 60.3% of wild type progeny became Dar (Figure 2A) although 94.65% remained infected as assessed by SYTO13 staining. These conditions allowed us to identify mutations and treatments that enhance the Dar phenotype.

When *tph-1(mg280)*, or another putative null allele *tph-1(n4622)*, animals were grown on lawns contaminated with 0.05% *M. nematophilum* we observed an increase in the percentage of progeny with the Dar phenotype, when compared to wild type controls (Figure 2A). On lawns contaminated with 10% *M. nematophilum* wild type controls, *tph-1(mg280)* and *tph-1(n4622)* progeny were all over 90% Dar (Figure 2A).

To determine whether the enhanced Dar phenotype of *tph-1(mg280)* and *tph-1(n4622)* was due to a decrease in serotonin synthesis we grew animals lacking *tph-1* on 0.05% *M. nematophilum* infection plates supplemented with exogenous serotonin. Treatment with exogenous serotonin was able to rescue the enhanced Dar response observed in *tph-1(mg280)* and *tph-1(n4622)* animals (Figure 2A), confirming that wild type levels of serotonin, synthesised by TPH-1, are required to suppress the wild type Dar response.

*C. elegans* uses behavioral avoidance strategies, as well as immune responses, to promote its survival in the presence of pathogens [6], [8], [13], [39]. *tph-1(mg280)* and *tph-1(n4622)* animals exhibit defects in avoidance of *Pseudomonas aeruginosa* [6], [14], [15] To analyse the role of serotonin synthesis in *M. nematophilum* avoidance behavior, a phenotype that is dependent on locomotion, we used the *tph-1(n4622)* allele, since the *tph-1(mg280)* allele has a background mutation that affects locomotion [40]. Over 70% of wild type animals were found outside bacterial lawns contaminated with *M. nematophilum* and this distribution was not altered in *tph-1(n4622)* animals (Figure S2A). Furthermore, both wild type and *tph-1(n4622)* animals showed a strong preference for *E. Coli* vs *M. nematophilum* in food choice assays (Figure S2B and C). Together our data indicates that serotonin signaling is not required for avoidance of *M. nematophilum*.

TPH-1 is expressed in the serotonergic neurons ADF, NSM and HSN and occasionally in AIM and RIH [16], [27]. To determine the site of action for TPH-1 in regulating the Dar phenotype we performed rescue experiments using a TPH-1 cDNA expressed from either the ADF-specific *srh-142* promoter [17], [41] or the *ceh-2* promoter that is expressed in NSM neurons [18], [42]. Expression of TPH-1 cDNA in ADF chemosensory neurons, but not in the neurosecretory motor neuron NSM, restored the number of Dar animals to wild type levels in *tph-1(mg280)* animals grown on lawns contaminated with 0.05% *M. nematophilum* (Figure 2B). TPH-1 cDNA expressed in both the ADF and NSM neurons of *tph-1(mg280)* animals did not enhance this rescue (Figure 2B). These results suggest that serotonin synthesis by TPH-1 in the ADF chemosensory neurons is able to inhibit the Dar phenotype in wild type animals.

### Serotonin synthesis in chemosensory neurons prolongs *M. nematophilum* colonization

We next asked how the Dar response is advantageous to infected *C. elegans* and whether this advantage could be suppressed by serotonin's effects on the Dar phenotype. *M. nematophilum* is found associated with the cuticle around the *C. elegans* rectal opening [19], [33] and the Dar phenotype may protect animals from severe infection by distorting the animal's anal region, allowing more rapid clearance of the pathogen from the rectum. Using the vital dye Syto13 to label *M. nematophilum* attached to the rectal opening, we monitored clearance of the pathogen from the rectum following infection. Wild type animals were able to clear more than 50% of the Syto13 labeled *M. nematophilum* within 90 minutes of transfer to plates without any bacteria (Figure 1F). Animals that had cleared the SYTO13 labeled *M. nematophilum* after 90 minutes remained Dar (Figure S3A–C), indicating that loss of pathogen from the rectal opening is not sufficient to reverse the Dar phenotype.

Many *dar-defective* mutants fail to show any SYTO13 staining in the presence of *M. nematophilum*, indicating that these genes are required for pathogen recognition and binding [3], [20]. However, a second class of *dar-defective* mutants remain SYTO13 positive [3], [21], [36]. This second class of genes act downstream of pathogen binding to trigger the Dar response to infection. Using this class of Dar-defective mutants *(mpk-1(ku1), unc-73(ce362)* and those described in this paper) we repeated our clearance assays. When Dar and Dar-defective animals were scored indifferently for the presence of SYTO13 labeled *M. nematophilum* we observed a significant decrease in the ability of these mutants to clear labeled pathogen when compared to wild type controls (R. McMullan and A. Anderson, data not shown), demonstrating that the Dar phenotype protects *C. elegans* during infection, at least in part, by increasing the rate of pathogen clearance.

Using this clearance assay we first asked whether exogenous serotonin treatment of wild type animals; which decreases the percentage of Dar animals, was able to alter the rate of pathogen clearance. Wild type animals treated with serotonin cleared the pathogen infection significantly more slowly than control animals (Figure 1F), indicating that exogenous serotonin is able to inhibit the immune response. The Dar-defective phenotype was observed in approximately 43% of wild type progeny when animals are infected in the presence of exogenous serotonin. To confirm that this Dar-defective phenotype was associated with slower pathogen clearance rates we repeated our clearance assay separating Dar and Dar-defective animals prior to SYTO13 labeling. Almost all animals were SYTO13 positive immediately following labeling and the rate of pathogen clearance in Dar animals was similar to that observed in untreated wild type animals (Figure S3D). However pathogen clearance rates were significantly slower in Dar-defective animals (Figure S3D) confirming the ability of exogenous serotonin to decrease the Dar phenotype results in inhibition of the immune response and decreased pathogen clearance.

To determine whether endogenous serotonin was also able to alter pathogen clearance rates we performed clearance assays using *tph-1(mg280)* and *tph-1(n4622)* animals lacking endogenous serotonin synthesis. Interestingly, although these animals show a wild type Dar response when infected with 10% *M. nematophilum* (Figure 2A) both *tph-1(mg280)* and *tph-1(n4622)* animals cleared SYTO13 labeled *M. nematophilum* significantly faster than wild type animals (Figure 2C). This is consistent with our observation that *tph-1(mg280)* and *tph-1(n4622)* animals grown on lawns contaminated with 0.05% *M. nematophilum* have a larger percentage of Dar animals than wild type controls and suggests that TPH-1 activity in wild type animals inhibits the immune response triggered by infection by *M. nematophilum*.

To determine whether the enhanced immune response of *tph-1(mg280)* and *tph-1(n4622)* was due to a decrease in serotonin synthesis we grew animals lacking *tph-1* on infection plates supplemented with serotonin. Treatment with exogenous serotonin was able to rescue the increased clearance rate observed in *tph-1(mg280)* and *tph-1(n4622)* animals (Figure 2C) confirming that endogenous levels of serotonin synthesis by TPH-1 are required to suppress the wild type immune response.

To determine where TPH-1 activity was required to reduce pathogen clearance rates we again expressed TPH-1 cDNA in either ADF or NSM neurons, or both. Expression of a TPH-1 cDNA in ADF chemosensory neurons, but not in the neurosecretory motor neuron NSM, was sufficient to rescue the rate of *M. nematophilum* clearance to wild type rates in *tph-1(mg280)* animals and this rescue was not enhanced when TPH-1 was expressed in both ADF and NSM (Figure 2D).

### Infection with pathogenic *M. nematophilum* does not alter expression of the serotonin biosynthetic enzyme TPH-1

**Figure 3:**
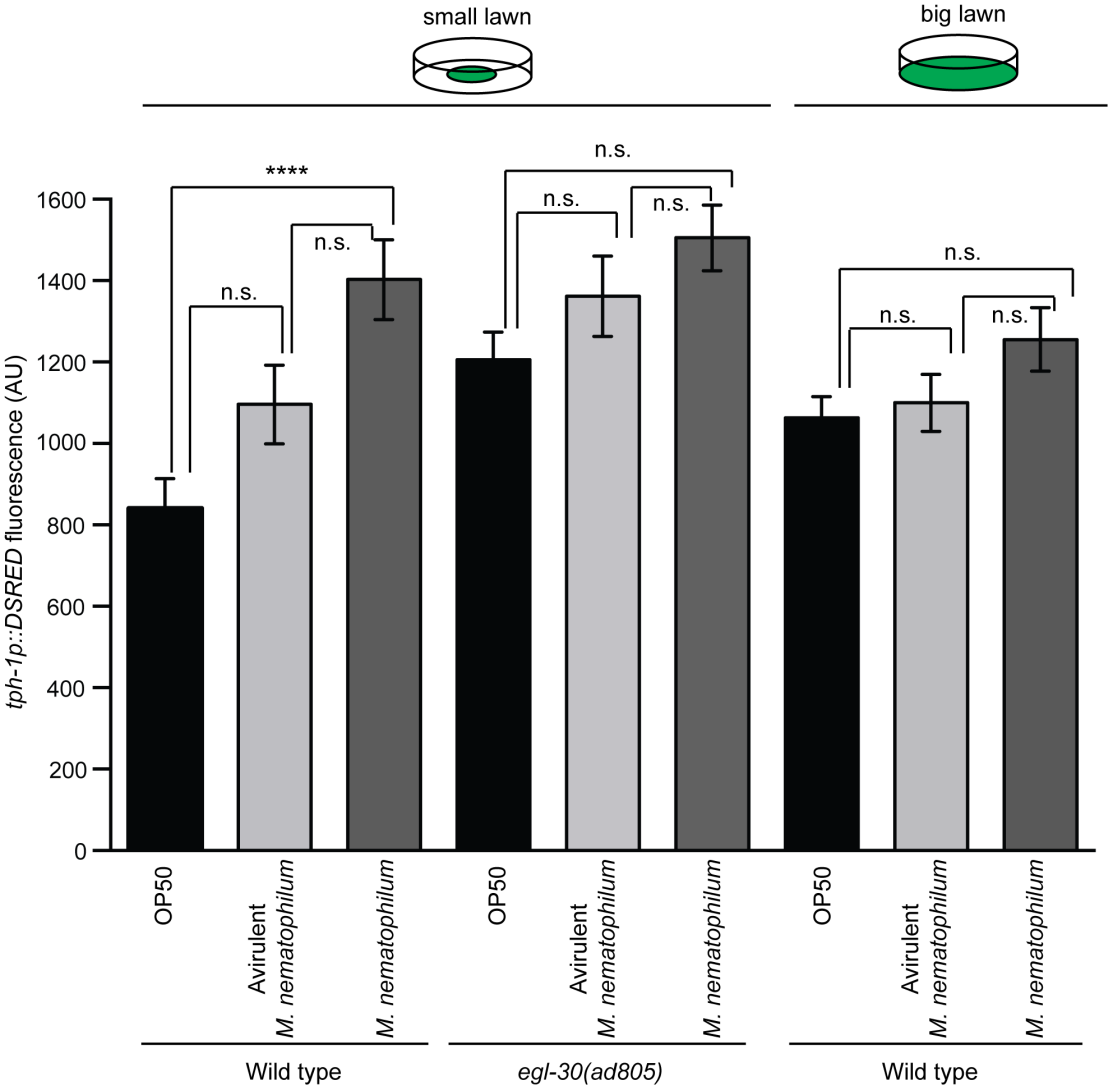
Increased expression of TPH-1 in ADF chemosensory neurons is caused by reduced contact with contaminated bacterial lawns. Wild type and *egl-30(ad805)* animals carrying an integrated *tph-1p*::DSRED transgene were infected with *M. nematophilum* or an avirulent form of *M. nematophilum* using standard (small lawn) or “big lawn” assay conditions. The mean *tph-1p*::DSRED fluorescence in ADF neurons was quantified. Expression of *tph-1p*::DSRED was significantly increased when wild type animals were grown on small lawns contaminated with virulent *M. nematophilum*. This increase in expression was not observed under conditions when animals were unable to leave the bacterial lawn; in *egl-30(ad805)* animals or when wild type animals were infected on “big lawns”.

Serotonin signaling in ADF chemosensory neurons suppresses the epithelial immune response to infection with *M. nematophilum*, raising the possibility that these neurons are able to sense and respond to the presence of pathogen by modifying their serotonin signaling. Serotonin levels in ADF can be altered by regulating transcription of *tph-1*, or via post-translational mechanisms that alter TPH-1 activity. Transcription of *tph-1* in ADF neurons is increased by exposure to pathogenic *Pseudomonas aeruginosa* PA14 [5], [6], [22], neuronal activity [23], [41] and heat stress [20], [43] and these changes can be monitored *in vivo* using strains expressing a fluorescent transgene under the control of the *tph-1* promoter. To determine whether infection with *M. nematophilum* altered transcription of *tph-1* we propagated animals stably expressing a *tph-1p::DSRED* transgene on small bacterial lawns contaminated with virulent, or avirulent, forms of *M. nematophilum*. Infection with virulent, or avirulent, forms of *M. nematophilum* did not alter the expression pattern of *tph-1p::DSRED* (data not shown) however infection increased expression levels of *tph-1p::DSRED* in ADF and NSM neurons relative to animals propagated on *E. Coli* alone (Figure 3 and Figure S4).

Wild type animals tend to avoid bacterial lawns contaminated with virulent *M. nematophilum* ([9], [24] and Figure S3) therefore it is possible that increased *tph-1p::DSRED* expression in the presence of *M. nematophilum* is caused by reduced contact with the bacterial lawn. To test this we first expressed the *tph-1p::DSRED* transgene in *egl-30(ad805)* animals that fail to avoid *M. nematophilum* contaminated lawns [36]. We did not observe any significant increases in *tph-1p::DSRED* expression in these *egl-30(ad805)* animals (Figure 3 and Figure S4). We also repeated our *tph-1p::DSRED* measurements using animals infected in a modified “big lawn” assay where they were unable to leave the food. Again we were unable to observed any significant increases in *tph-1p::DSRED* expression when animals were unable to avoid *M. nematophilum* contaminated lawns (Figure 3 and Figure S4). Together these results indicate that the changes in TPH-1 expression levels we observed were largely due to reduced contact with the bacterial lawn when it was contaminated with virulent *M. nematophilum* and not due to infection.

### The Gαo RGS EGL-10 acts in the rectal epithelium to modify the immune response and affect pathogen clearance

Several receptors have been identified that bind serotonin in *C. elegans*, including a serotonin gated chloride channel (MOD-1) and several G-protein coupled receptors (GPCRs) (SER-1, SER-4 and SER-7) [25], [44]–[46]. Infection of *mod-1(ok103), ser-1(ok345), ser-7(tm1325), ser-7(ok1944), ser-4(ok512)* mutants and *ser-1(ok345);ser-7(tm13325)* double mutants indicates that at least two GPCRs (SER-1 and SER-7) are required for serotonin effects on the immune response (Figure S5).

GPCRs activate intracellular signaling via specific G-proteins. In *C. elegans* the effects of serotonin on neuronal activity at the neuromuscular junction are mediated by the G-protein GOA-1(Gαo) [26], [47]. Therefore we asked whether GOA-1(Gαo) signaling was also required for serotonin-mediated inhibition of the immune response. We first infected several available *goa-1*(Gαo) mutants (*goa-1(sa734)*, *goa-1(n1134)* and *goa-1(n363)*) with *M. nematophilum* as adults or larvae to determine whether loss of GOA-1(Gαo) signaling altered the Dar phenotype. For reasons that we were unable to determine, the majority of these animals arrested at the L1/L2 larval stage and failed to reach adulthood. Therefore we were unable to score the Dar phenotype of these animals (data not shown).

**Figure 4:**
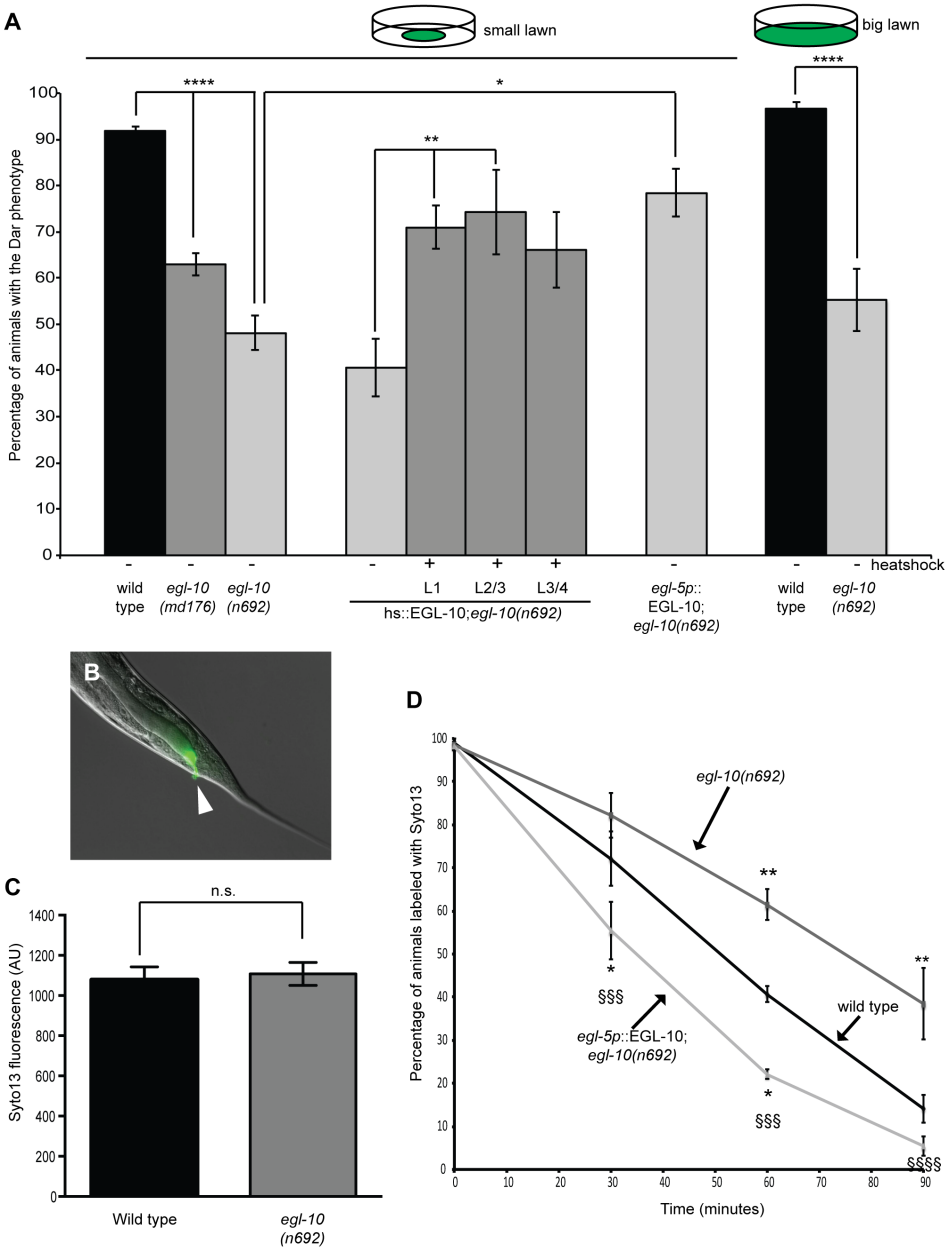
The Gαo RGS EGL-10 is required in rectal epithelial cells to regulate the immune response and affect pathogen clearance. *egl-10* mutants were infected with *M. nematophilum* using either standard assay conditions (small lawn) or conditions where animals were unable to avoid the pathogen (big lawn) and the percentage of Dar progeny scored. *egl-10(n692)* and *egl-10(md176)* significantly decreased the percentage of Dar animals (A). Expression of EGL-10 cDNA, using a heatshock-inducible promoter, at L1, L2/L3 and L3/L4 stage (hs::EGL-10), or a rectal epithelial promoter (*egl-5p*::EGL-10), rescued the Dar phenotype in *egl-10(n692)* animals (A). Although *egl-10(n692)* animals failed to produce a wild type Dar response similar amounts of *M. nematophilum*, labeled using the nucleic acid stain SYTO13, still attached to the anal opening (indicated with an arrow in B) (B and C). The rate of clearance of SYTO13 labeled pathogen was significantly decreased in *egl-10(n692)* animals and this was rescued by expression of EGL-10 cDNA in the rectal epithelium which cleared labeled pathogen more rapidly than wild type animals (D). In D * indicates significance relative to wild type. § indicates significance relative to *egl-10(n692)*.

Genetic and biochemical experiments have shown that the conserved Regulator or G-protein signaling (RGS) protein, EGL-10, is a specific inhibitor of GOA-1(Gαo) activity [26], [48]. Therefore we used animals carrying null mutations in *egl-10* to increase *goa-1*(Gαo) signaling indirectly, mimicking the effects of too much serotonin signaling. When *egl-10(md176)* and *egl-10(n692)* adults were infected with *M. nematophilum* we observed a significant decrease in the percentage of progeny exhibiting the Dar phenotype (Figure 4A). However similar levels of SYTO13-labeled *M. nematophilum* were still observed adhering to the rectum of these animals indicating that increased GOA-1(Gαo) signaling did not alter the ability of the pathogen to attach to the cuticle (Figure 4B and C). Furthermore, this decrease in the Dar phenotype was not caused by increased behavioral avoidance of the pathogen because the Dar phenotype was still decreased when *egl-10(n692)* were infected using a modified ‘big-lawn’ assay where they were unable to avoid the pathogen (Figure 4A). Consistent with this decrease in the Dar phenotype we observed that *egl-10(n692)* mutants cleared pathogen infections from their rectal opening more slowly than wild type (Figure 4D), indicating that activating GOA-1(Gαo) is able to suppress the immune response.

The Dar phenotype requires activation of multiple signaling pathways in the *C. elegans* rectal epithelium [27], [35], [36]. To determine the site of action for EGL-10 (and therefore GOA-1(Gαo)) in the immune response we performed rescue experiments using EGL-10 cDNA expressed in the rectal epithelial cells, using a 1.3 Kb *egl-5* promoter fragment [28], [49]. Expression of EGL-10 cDNA in the rectal epithelium was sufficient to rescue the defective Dar phenotype in *egl-10(n692)* animals (Figure 4A). In addition rectal epithelial expression of EGL-10 rescued the slow clearance of pathogen in *egl-10(n692)* animals. Indeed these animals cleared labeled pathogen faster than wild type animals (Figure 4D) suggesting that overexpression of EGL-10 cDNA in these transgenic animals was able to decrease GOA-1(Gαo) activity and indicating that GOA-1(Gαo) signaling acts in the rectal epithelial cells of wild type animals to inhibit the immune response. To confirm that EGL-10 was required for the Dar response to infection rather than development of the rectal epithelium we also performed rescue experiments in *egl-10(n692)* mutants using a heat shock inducible EGL-10 cDNA transgene. We were able to partially rescue the Dar phenotype in *egl-10(n692)* mutants by expressing EGL-10 cDNA 10–18 hours prior to adulthood (L3/L4 larval stage), indicating that GOA-1(Gαo) signaling in adult animals is required for this response (Figure 4A).

### Rectal epithelial EGL-10 signaling acts downstream of neuronal serotonin signaling to modify the immune response and affect pathogen clearance

**Figure 5:**
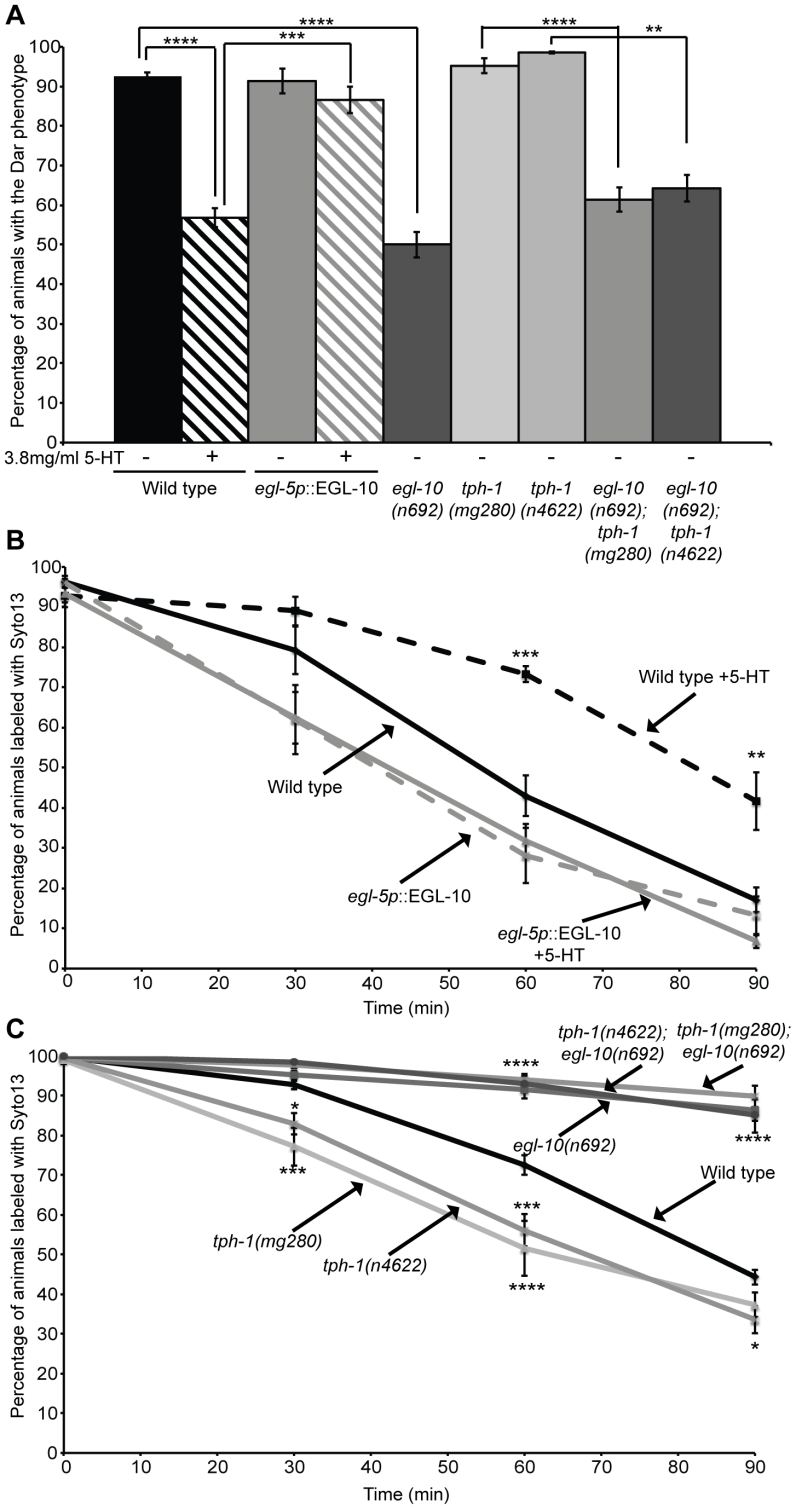
Rectal epithelial EGL-10 acts downstream of serotonin to modify the immune response and affect pathogen clearance. Treatment of wild type animals with 3.8/ml 5-HT caused a decrease in the number of Dar animals following infection with *M. nematophilum* (A) and decreased the clearance of SYTO13 labeled pathogen from the rectal opening (B). Serotonin was unable to decrease the percentage of Dar animals (A) or the rate of pathogen clearance (B) when EGL-10 cDNA was overexpressed in the rectal epithelium of wild type animals suggesting that GOA-1(Gáo) signaling in the rectal epithelium is required for serotonin to suppress the immune response. Animals lacking *tph-1* have wild type levels of Dar response on lawns contaminated with 10% *M. nematophilum* (A) but are more able to clear pathogen infections than wild type (C). Conversely activation of GOA-1(Gáo) using *egl-10* loss-of-function mutants results in a decrease in the percentage of Dar animals (A) and infections clear more slowly than wild type animals (C). To determine whether GOA-1(Gáo) acts downstream of serotonin we combined *egl-10(n692)* with *tph-1(mg280)* or *tph-1(n4622)*. The percentage of Dar animals (A) and the rate of pathogen clearance was indistinguishable between *egl-10(n692)* and these double mutants (C). Thus GOA-1(Gáo) signaling acts downstream of serotonin synthesis to suppress the immune response to *M. nematophilum* infection.

Animals lacking GOA-1(Gαo) are resistant to the effects of exogenous serotonin treatment on egg laying and locomotion [29], [30], [47], [50]. To determine whether serotonin inhibits the immune response via a GOA-1(Gαo) signaling pathway we first treated adult animals with decreased rectal epithelial GOA-1(Gαo) signaling, (by overexpressing the RGS, EGL-10 in the rectal epithelial cells of wild type animals), with exogenous serotonin and scored the Dar phenotype of their progeny. Addition of exogenous serotonin during *M. nematophilum* infection suppressed the Dar phenotype of wild type animals but not those overexpressing EGL-10 (Figure 5A). Furthermore exogenous serotonin was no longer able to reduce the rate of pathogen clearance when EGL-10 was overexpressed in rectal epithelial cells (Figure 5B). Together these data indicate that wild type levels of GOA-1(Gαo) signaling in the rectal epithelium are required for serotonin's inhibitory effects on the immune response.

We also asked whether increasing GOA-1(Gαo) signaling, using *egl-10(n692)* mutants, was able to rescue the higher pathogen clearance rate we observed in *tph-1* mutant animals unable to synthesis serotonin. Pathogen clearance rates in *tph-1(mg280);egl-10(n692)* and *tph-1(n4622);egl-10(n692)* double mutants were indistinguishable from *egl-10(n692)* animals (Figure 5C). We also observed a significant decrease in the Dar phenotype in these double mutants that was similar to the decrease observed in *egl-10(n692)* (Figure 5A) further indicating that GOA-1(Gαo) acts downstream of serotonin to inhibit the immune response.

**Figure 6:**
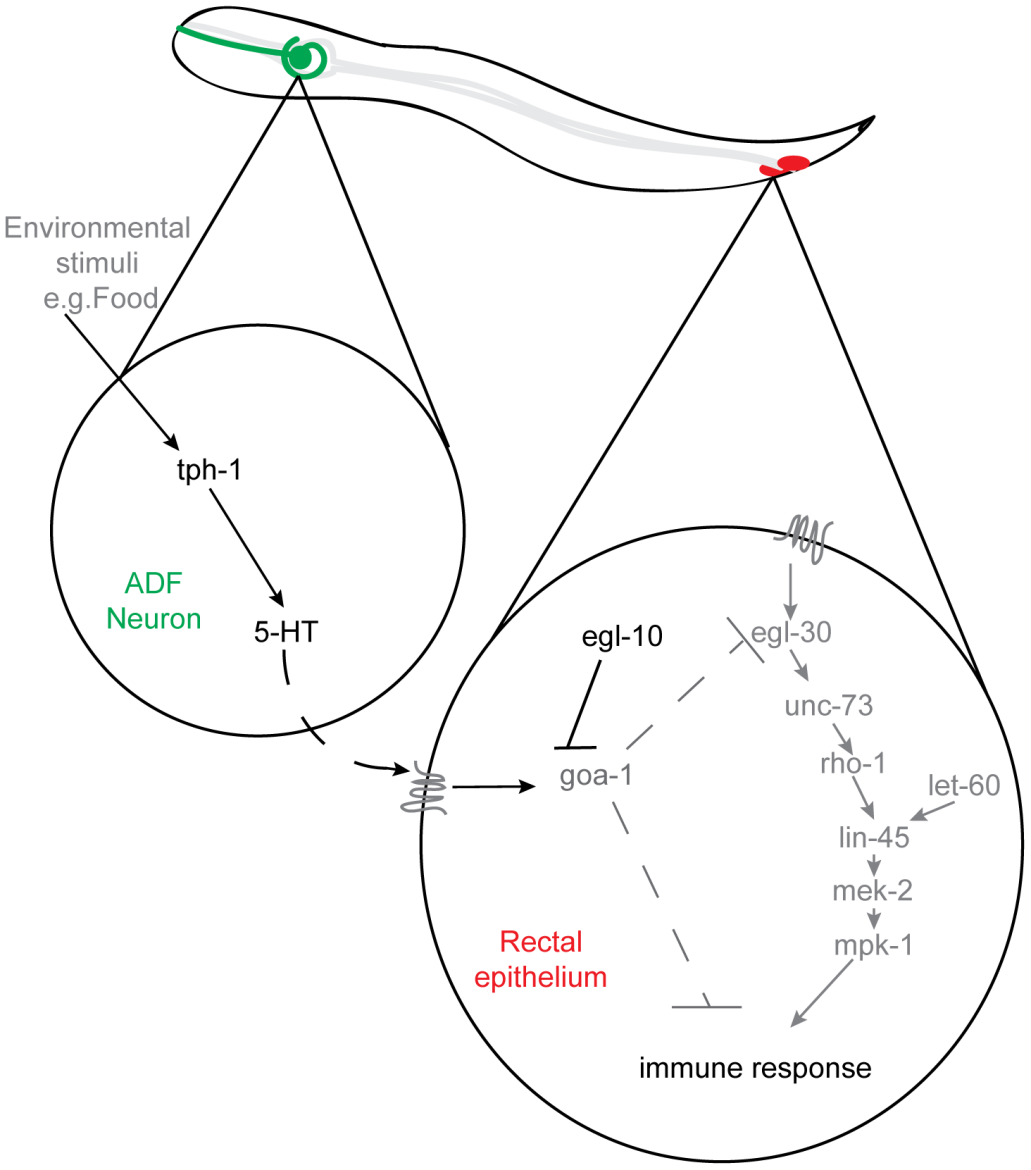
Serotonin synthesis in chemosensory neurons inhibits the immune response by altering rectal epithelial G-protein signaling. In response to environmental cues, such as the presence or absence of food, serotonin, released from ADF chemosensory neurons acts, directly or indirectly, to regulate GOA-1(Gαo) signaling in the rectal epithelium. This signaling suppresses the Dar phenotype that forms part of the innate immune response and limits the rate of pathogen clearance from the rectal opening.

Taken together our data indicates that serotonin synthesized in ADF chemosensory neurons acts via GOA-1(Gαo) signaling in the rectal epithelium to suppress the immune response (Figure 6).

### Serotonin signaling acts upstream of, or in parallel to, the EGL-30(Gαq) pathway to regulate the immune response

The Dar phenotype requires activation of multiple signaling pathways in the *C. elegans* rectal epithelium [30], [35], [36]. We have previously shown that signaling via the G-protein EGL-30(Gαq) is required for the Dar response to infection with *M. nematophilum* [36]. To determine whether serotonin signaling acts in this EGL-30(Gαq) pathway, or a distinct pathway, we used transgenic animals overexpressing a constitutively active form of EGL-30(Q205L) (Gαq) in the rectal epithelium (RE::EGL-30*). When this transgene is expressed in wild type animals approximately 49% of them develop the Dar phenotype in the absence of infection (Table 1 and [36]) although all animals expressing this transgene are still able to trigger the Dar phenotype when infected with *M. nematophilum* (data not shown).

**Table 1:** Analysis of genetic interactions between the serotonin – GOA-1(Gαo) and EGL-30(Gαq) signaling pathways.

Effect of decreased serotonin signaling on EGL-30*-induced Dar		
Genotype	% animals with the Dar phenotype ± s.e.m.	n value
RE::EGL-30*	49.5±1.9	36
RE::EGL-30*;*tph-1(mg280)*	78.9±1.5^a^	4
RE::EGL-30*;*tph-1(mg280)*; ADF::TPH-1	49.9±2.7^b^	3
RE::EGL-30*;*tph-1(mg280)*; NSM::TPH-1	76.2±1.5^c^	3
RE::EGL-30*;*tph-1(mg280)*;ADF::TPH-1; NSM::TPH-1	55.6±0.7^d^	4
RE::EGL-30*;*tph-1(n4622)*	76.5±2.1^a^	9
RE::EGL-30*+50 µg/ml methiothepin	76.5±1.8^a^	11
**Effect of decreased serotonin signaling on the Dar phenotype of ** ***egl-30(ad805)***		
Wild type on 10% *M. nematophilum*	92.5±1.0	45
*tph-1(n4622)* on 10% *M. nematophilum*	94.7%±0.4	3
*egl-30(ad805)* on 10% *M. nematophilum*	4.9±1.4^e^	3
*tph-1(n4622);egl-30(ad805)* on 10% *M. nematophilum*	4.9±2.0^e^	3
Effect of increased serotonin signaling on EGL-30*-induced Dar		
RE::EGL-30*+3.8 mg/ml serotonin	51.6±3.5	8
RE::EGL-30*;*egl-10(n692)*	49.5±1.7	5
Effect of increased serotonin signaling on RHO-1*- and LIN-45*-induced Dar		
RE::RHO-1*	86.5±7.0	4
RE::RHO-1*+3.8 mg/ml serotonin	85.0±4.0	13
RE::RHO-1*;*egl-10(n692)*	96.0±1.8	5
RE::LIN-45*	98.1±1.3	6
RE::LIN-45*+3.8 mg/ml serotonin	95.7±2.6	5
RE::LIN-45*;*egl-10(n692)*	95.1±1.3	8

The number of Dar animals was scored as a percentage of the total. Drug treatments were as described in the Methods. Values are means +/− the standard error. No Dar animals were observed in uninfected *tph-1(mg280)*, *tph-1(n4622)* or *egl-10(n692)* single mutants.

a = p≤0.0001 relative to RE:::EGL-30*.

b = p≤0.01 relative to RE::EGL-30*;*tph-1(mg280)*.

c = p≤0.001 relative to RE:::EGL-30*.

d = p≤0.05 relative to RE::EGL-30*;*tph-1(mg280)*.

e = p≤0.0001 relative to wild type on 10% *M. nematophilum*.

We asked what effect blocking serotonin signaling, in two different ways, had on the Dar phenotype caused by RE::EGL-30*. Firstly, we blocked serotonin signaling by treating uninfected RE::EGL-30* animals with the serotonin receptor antagonist methiothepin. Treatment of adult RE::EGL-30* animals with methiothepin increased the percentage of Dar progeny from 49.5% to 76.5% (Table 1). Secondly, we expressed RE::EGL-30* in *tph-1(mg280)*, or *tph-1(n4622)*, animals in the absence of infection. We observed an increase the percentage of Dar animals from 49.5% in wild type animals to 75.3% in *tph-1(mg80)* and 76.5% in *tph-1(n4622)* animals (Table 1). This increase could be rescued by expression of the TPH-1 cDNA in ADF, but not NSM, neurons of *tph-1(mg280)* (Table 1). These results support our previous observations that blocking serotonin signaling increases the Dar phenotype when animals are infected with 0.05% *M. nematophilum*. We also asked what effect blocking serotonin signaling had on the Dar phenotype of infected *egl-30(ad805)* animals. These animals are strongly Dar-defective [36] and we did not observe any significant differences in the Dar phenotype of *egl-30(ad805)* animals and the *tph-1(n4622);egl-30(ad805)* double mutant (Table 1). Taken together these results indicate that serotonin is acting either downstream of, or in parallel to, the EGL-30(Gαq) signaling pathway.

We next asked what effect activating serotonin signaling would have on the Dar phenotype triggered by RE::EGL-30*. To do this we grew uninfected RE::EGL-30* animals in the presence of exogenous serotonin for at least one generation. Serotonin treatment did not significantly alter the number of Dar animals in this transgenic strain (Table 1). EGL-30(Gαq) activates a Rho GEF TRIO – RHOA - RAF signaling pathway to trigger the Dar phenotype in response to infection and activation of downstream components of this pathway in the rectal epithelium; using cell-specific overexpression of constitutively active forms of RHO-1(G14V) (RhoA) (RE::RHO-1*) or LIN-45(S312A,S453A) (Raf) (RE::LIN-45*), also results in the Dar phenotype in the absence of infection. We observed no significant decrease in the number of Dar animals when we grew uninfected RE::RHO-1* or RE::LIN-45* animals in the presence of exogenous serotonin for at least one generation (Table 1). This data most strongly supports the hypothesis that serotonin acts upstream of the EGL-30(Gαq) signaling pathway during this response.

During neurotransmission the EGL-30(Gαq) pathway acts antagonistically to GOA-1(Gαo) [47], [51] with GOA-1(Gαo) reported to act upstream of, or in parallel to EGL-30(Gαq) [51], [52]. Our data suggests that this antagonism also exists in the *C. elegans* rectal epithelium where it plays an important role in modulating the immune response. Therefore we asked whether GOA-1(Gαo) and EGL-30(Gαq) act in the same, or parallel, pathways by expressing RE::EGL-30* in *egl-10(n692)* animals. Activation of GOA-1(Gαo) signaling in *egl-10(n692)* did not significantly alter the number of RE::EGL-30*-induced Dar animals relative to wild type animals (Table 1). Furthermore, when two other transgenes that cause the Dar phenotype (RE::RHO-1* and RE::LIN-45*) were expressed in uninfected *egl-10(n692)* animals the percentage of Dar animals was not significantly different from that observed in wild type animals expressing these transgenes (Table 1). This data supports the model that the serotonin - GOA-1(Gαo) pathway acts upstream of, or in parallel to, the EGL-30(Gαq) pathway to regulate the immune response (Figure 6).

## Discussion

### *C. elegans* uses neurotransmitters to influence the immune response

Insulin-like neuropeptides and cytokines, produced in *C. elegans* neurons, can influence its ability to mount an immune response to pathogen infection [4], [7]. In mammals a number of neurotransmitters can also influence the immune response [13] however the regulation of *C. elegans* immunity by neurotransmitters remains largely unexplored. Although Sun et. al. [12] have demonstrated that signaling downstream of the neurotransmitter octopamine suppresses the immune response to *P. aeruginosa* infection, the function of neurotransmitters themselves remains undetermined. Here we show that the classical neurotransmitter, serotonin, suppresses the immune response to infection with the pathogen *M. nematophilum*. Serotonin synthesized in chemosensory neurons acts, via at least two serotonin GPCR's, to regulate G-protein signaling in rectal epithelial cells and suppress the Dar phenotype. This leads to a reduction in the animal's ability to clear the pathogen infection.

This is the first demonstration of a role for serotonin signaling in regulation of a *C. elegans* immune response, however serotonin regulates both innate and adaptive mammalian immune responses [14], [15] suggesting further parallels between *C. elegans* and mammalian immunity.

Interestingly, although serotonin does not appear to modify immune responses triggered by infection with other pathogens, it does alter *C. elegans* behavioral response to these pathogens. When animals are exposed to *Pseudomonas aeruginosa* PA14 they modify their olfactory preferences so that they learn to avoid these pathogenic bacteria [6]. This learnt avoidance results in increased survival following infection [5] and requires serotonin signaling in the same chemosensory neuron required to suppress the immune response to *M. nematophilum* [6], Although *C. elegans* avoids lawns contaminated with *M. nematophilum* we did not observed any role for serotonin signaling in this behavioral response. Thus serotonin signaling in the same neuron can trigger distinct responses, requiring different target cells, depending on the environment *C. elegans* encounters. It is possible that different levels of serotonin are required to trigger these different responses. Alternatively signaling in other neurons may be regulated by infection. Changes in the activity of these neurons may modify how downstream target cells respond to the presence of serotonin.

### Environmental signals alter the immune response by influencing serotonin signaling from chemosensory neurons

Although *C. elegans* neurons have been shown to produce insulin-like neuropeptides and cytokines that influence the immune response [4], [7] the identity of these neurons has remained largely elusive. Here we identify a single neuron that influences *C. elegans* immunity.

In the *C. elegans* adult hermaphrodite only ADF, NSM, HSN, AIM, RIH and VC4/5 neurons have been shown to contain serotonin [28], [53], [54] and the tryptophan hydroxylase TPH-1, required to synthesis serotonin, is only expressed in ADF, NSM and HSN [27], suggesting that AIM, RIH and VC4/5 take up serotonin synthesized by other neurons. Using cell specific rescue experiments we have shown that expression of TPH-1 (and therefore serotonin synthesis) in ADF is required to suppress the immune response to infection by *M. nematophilum*.

ADF neurons are a set of two bilaterally symmetrical chemosensory neurons that contact the external environment via sensory cilia in the amphid [55], raising the possibility that chemical cues present in the environment can alter ADF activity and influence the immune response. What are the environmental signals that trigger ADF activation leading to serotonin synthesis and suppression of the immune response following *M. nematophilum* infection?

One possibility is that chemical cues produced by the pathogen itself are detected by ADF. *Pseudomonas aeruginosa* infection increases TPH-1 expression [5], [6] and stimulates ADF neuronal activity [56] to promote behavioral avoidance of the pathogen [6]. In contrast *M. nematophilum* does not appear to regulate serotonin synthesis in ADF, because we only observed changes in TPH-1 expression under conditions where there was decreased contact with the *M. nematophilum* contaminated bacterial lawn. However, we cannot exclude the possibility that other aspects of ADF activity, including regulation of TPH-1 activity or serotonin release, are regulated by the pathogen. Regardless of how infection with *M. nematophilum* regulates serotonin signaling, suppression of this pathway alone is not sufficient to trigger the Dar phenotype. We did not observe the Dar phenotype in uninfected *tph-1* mutants or in wild type animals treated with methiothepin (R. McMullan and A. Anderson, unpublished observation) indicating that infection with *M. nematophilum* must regulate additional signaling pathways (including the EGL-30(Gαq) pathway) to trigger the immune response.

Another environmental cue that may alter serotonin synthesis to suppress the immune response is the presence of food. In *C. elegans* serotonin signals the presence of food [28], and because ADF neurons directly contact the environment it has been suggested that they may couple environmental food signals with serotonergic neurotransmission [54]. In support of this we observed that *tph-1* mutant animals (that behave as if they were starved in the presence of food), were able to respond to lower levels of *M. nematophilum* infection than wild type animals. We have also obtained anecdotal evidence that infection causes starved animals to become Dar more easily than well-fed animals (R. McMullan and A. Anderson, unpublished observation) suggesting that the presence (or absence) of food is able to influence the immune response. Furthermore serotonin signaling is able to suppress the Dar response when it is triggered in the absence of infection (using RE::EGL-30*) because decreasing serotonin synthesis (using *tph-1* mutants), or signaling (using methiothepin), both increased the percentage of Dar positive RE::EGL-30* animals. One possible explanation for these observations is that in the presence of food, wild type levels of serotonin inhibit the Dar phenotype. It is possible that the presence of food is sensed by ADF, increasing serotonin synthesis and suppressing the immune response. In this way *C. elegans* would be able to integrate several environmental signals including the availability of food and the presence of pathogenic microbes and respond accordingly.

### Serotonin synthesized in *C. elegans* head neurons acts on distant target cells in the tail

We have shown that serotonin synthesized and released from the amphid chemosensory neuron ADF in the animal's head acts on rectal epithelial cells located in the animal's tail. How does serotonin released from these cells influence signaling in distant target cells in the tail?

ADF forms synapses with 17 other interneurons and sensory neurons and gap junctions with an additional two sensory neurons (www.wormweb.org). The rectal epithelial cells, where GOA-1(Gαo) signaling is required to suppress the immune response, have not been reported as postsynaptic targets of ADF suggesting that serotonin is not released directly onto these cells. In principle it is possible that infection of *C. elegans* with *M. nematophilum* alters the connectivity of ADF such that the rectal epithelium become a postsynaptic target however we did not observe any gross changes in the expression pattern of a *tph-1p::DSRED* reporter, that is expressed in ADF neurons, following *M. nematophilum* infection (data not shown) and it seems unlikely that infection would cause such dramatic reorganization of the nervous system. Alternatively, serotonin may diffuse from its release sites to act on distant cells. This is consistent with previous *C. elegans* work showing that serotonin released from ADF travels extrasynaptically to RIH and AIM interneurons where it accumulates using the serotonin transporter mod-5 [54]. Extrasynaptic serotonin signaling is conserved in the vertebrate brain [57] and may also contribute to serotonin regulation of the mammalian immune response as B cells take up serotonin released from the noradrenergic neurons that innervate lymphatic tissue [58].

Another possibility is that serotonin does not act directly on the rectal epithelial cells. Several studies have placed GOA-1(Gαo) signaling downstream of serotonin in the regulation of locomotion [47], [52]. These studies have defined the site of action for GOA-1(Gαo) as the cholinergic motor neurons located in the ventral nerve cord [47]. Until recently, it remained unclear whether serotonin acts directly on serotonin receptors expressed on these cells to influence GOA-1(Gαo) signaling or whether serotonin simulates interneurons to release signals that activate GOA-1(Gαo) – coupled GPCRs on these motor neurons. Work by Gürel et. al. [59] identifies the serotonin receptors required for control of locomotion as MOD-1 and SER-4. SER-4 and MOD-1 expression were detected in a non-overlapping subset of head and tail interneurons while MOD-1 expression was also found in GABAergic motor neurons located in the ventral nerve cord indicating that serotonin must act indirectly on cholinergic motor neurons to regulate GOA-1(Gαo) signaling and locomotion [59]. Could this also be the case for serotonin regulation of the immune response? At least two serotonin GPCRs; SER-1 and SER-7, mediate the effect of exogenous serotonin on the immune response however expression of these receptors has not been reported in the rectal epithelium. Perhaps *M. nematophilum* infection alters the expression of these receptors, and in the future determining the expression pattern of these receptors during infection will address serotonin's mechanism of action.

### A conserved G-protein signaling network acts in neurons and epithelial cells to elicit different responses

In *C. elegans* cholinergic motor neurons a network of G-proteins regulates acetylcholine release to alter locomotion [60]. EGL-30(Gαq) and GOA-1(Gαo) act antagonistically to regulate acetylcholine release and control locomotion [51]. *egl-30* mutants have decreased acetylcholine release resulting in slower locomotion rates [51] while in *goa-1* mutants acetylcholine release and locomotion rates are increased [47]. Animals lacking *goa-1* are resistant to the effects of serotonin on locomotion suggesting that slowing responses, triggered by serotonin in the presence of food, may be mediated by GOA-1(Gαo) signaling in cholinergic neurons [47]. We have previously shown that EGL-30(Gαq) is required in the rectal epithelium for the immune response to infection by *M. nematophilum* [36] and here we show that GOA-1(Gαo) acts antagonistically to EGL-30(Gαq) in these cells to suppress the immune response. Our data demonstrate that the same G-protein network is activated in different tissues (neurons and epithelial cells), to elicit different responses (locomotion and immunity).

In neurons, genetic data places GOA-1(Gαo) and EGL-30(Gαq) in parallel pathways [51] however GOA-1(Gαo) may regulate the activity of the RGS EAT-16, which inactivates EGL-30(Gαq), placing GOA-1(Gαo) signaling upstream of EGL-30(Gαq) [61]. Our genetic data suggests that GOA-1(Gαo) acts either upstream of, or in parallel to, EGL-30(Gαq) in the immune response. The Dar response provides an opportunity to study the interactions between these pathways in a new context and it will be interesting to determine whether the immune response is altered in *eat-16* mutants.

Although serotonin acts upstream of G proteins in both neurons and epithelial cells, G-protein signaling activates different downstream signaling pathways in each of these cell types. In neurons EGL-30(Gαq) and GOA-1(Gαo) act antagonistically to control levels of the second messenger diacylglycerol (DAG) [47], [51] however DAG is not required to alter rectal epithelial cell shape and size and trigger the Dar phenotype (R. McMullan, unpublished observation). Conversely the ERK MAP Kinase pathway is required downstream of EGL-30(Gαq) in epithelial cells to trigger that Dar phenotype but does not appear to affect acetylcholine release [36].

### *C. elegans* as a model to study bidirectional cross talk between neurotransmitters and the immune response

Alterations in serotonin signaling have been implicated in multiple neurological disorders including anxiety, depression, autism and Alzheimer's disease [62]–[64]. There is growing evidence of altered immune function in these disorders that may contribute to their pathology (reviewed in [15]). Indeed in 1991 Smith proposed the macrophage theory of depression suggesting that excessive secretion of cytokines caused depression by altering serotonin levels in the brain [65]. Although it is not possible to ascertain the psychological state of model organisms such as *C. elegans* they have proved extremely useful in investigating the conserved molecular mechanisms that underlie changes in serotonin signaling in response to the environment. We have yet to determine whether the *C. elegans* immune response is able to reciprocally regulate serotonin signaling however analysis of serotonin-regulated behaviors following infection should begin to address this question. Our work demonstrates that *C. elegans* can also be used as a model to study the reciprocal cross talk between neurotransmitters and the immune response that may be important for the pathology of disorders such as depression.

## Materials and Methods

### Strains

*C. elegans* strains used in this study are detailed in Table S1. Gene ID's for genes used are detailed in Table 2. All strains were cultivated at 20°C on nematode-growth media (NGM) plates seeded with *E. Coli* OP50, unless otherwise stated, and maintained as described previously [66]. Where indicated Methiothepin (50 µg/ml) or Serotonin creatine sulfate (3.8 mg/ml) was added to NGM before pouring and plates were used within 5 days. Unless indicated adult animals were transferred to drug plates and their progeny were scored.

**Table 2:** Gene ID numbers from WormBase (www.wormbase.org).

	Gene ID
TPH-1	WBGene00006600
EGL-30	WBGene00001196
EGL-10	WBGene00001179
GOA-1	WBGene00001648
RHO-1	WBGene00004357
LIN-45	WBGene00003030
SER-1	WBGene00004776
SER-4	WBGene00004779
SER-7	WBGene00004780
MOD-1	WBGene00003386
LIN-48	WBGene00003033

### Transgenes and germline transformation

Plasmids (listed as pRJM) were constructed using standard techniques, and verified by sequencing. Transgenic strains (listed as *impEx*) were isolated by microinjection of the plasmid together with *acr-2::gfp* (a gift of J. Kaplan, Massachusetts General Hospital) or *unc-17::gfp* (a gift of S. Nurrish, University College London, UK) at 50 ng/µl as a marker. In all experiments matched animals not expressing the injection marker were assayed in parallel as a control. Data was only included if the phenotype of non-transgenic animals was comparable to that of the parental strain.

### EGL-10 transgenes

The wild type EGL-10 cDNA was isolated from wild type N2 RNA using standard techniques and verified by sequencing. This cDNA was subcloned into either the pPD49_78 heat shock vector (a gift of A. Fire Stanford University CA) (pRJM174) or a vector driving expression from a 1.3 Kb *egl-5* promoter fragment that drives GFP expression in B, K, F, U, P12.pa and three body wall muscles in the posterior [49] (pRJM176). These plasmids were injected at 20 ng/µl into *egl-10(n692)*. *impEx031* and *impEx020* contain extrachromosomal versions of pRJM174 and pRJM176 respectively. *impEx031;egl-10(n692)* were backcrossed to remove the *egl-10(n692)* mutation in order to obtain animals overexpressing EGL-10 in the rectal epithelium.

### Food choice assay

Assays were performed essentially as described in McMullan et. al. [36] with the following changes. OP50 or CBX102 bacterial cultures were grown to the same optical density in LB and 40 µl was placed on opposite sides of a 60 mm NGM plate. One-day old adult animals were transferred to NGM plates lacking food for 30 minutes and then washed in M9 and allowed to settle before aspiration. A suspension of animals in a drop of M9 was placed equidistant from each bacterial lawn, numbers of animals varied from 25 to 100. Choice index = (number of animals on lawn A- number of animals on lawn B)/number of animals on lawn A+B. In all experiments lawn A was OP50 and B was CBX102 *M. nematophilum*. Experiments were performed in triplicate and repeated at least three times.

### *M. nematophilum* infection

Infection with *M. nematophilum* was performed as described previously [36]. NGM plates were seeded with either 10% or 0.05% *M. nematophilum* diluted in OP50 *E. Coli*. Unless otherwise stated adult animals were transferred from OP50 plates to infection plates and maintained at 20°C. F1 progeny were scored for the presence or absence of the Dar phenotype once they reached L4 or adult stages. In the case of hs::EGL-10;*egl-10(n692)* animals (Figure 4A) and wild type animals in Figure 1B synchronized populations of L1 animals were obtained by bleaching. These animals were transferred to infection plates as L1's or grown on standard *E. Coli* OP50 plates for 24 (L2/L3 stage), or 48 hours (L3/L4 stage), before transferring to infection plates. This generation was assayed for the presence of the Dar phenotype when animals reached L4 or adult stages. Between 30 and 50 animals were scored per plate. Experiments were performed in triplicate and repeated at least three times.

For experiments using exogenous serotonin plates were prepared as described above and were seeded with 10% or 0.05% *M. nematophilum* diluted in OP50 *E. Coli*. Plates were used within 5 days. Animals were not pretreated with serotonin prior to *M. nematophilum* infection and serotonin was present throughout infection with *M. nematophilum*.

For imaging experiments the avirulent *M. nematophilum* strain UV336 [67] was used and plates were prepared identically to plates seeded with virulent, CBX102, *M. nematophilum*.

SYTO13 staining was performed as described previously [35]. Following incubation with SYTO13 animals were either transferred to unseeded plates, for clearance assays as described below, or mounted for imaging.

### Clearance of Syto13 labeled *M. nematophilum*

Animals were infected with *M. nematophilum* as described above and SYTO13 labeling was performed as described previously [35] except that after a 60 minute incubation with SYTO13 10–20 µl of settled, stained worms were transferred to NGM plates lacking food. After drying the number of animals colonized by SYTO13 positive *M. nematophilum* was scored using a Nikon SMZ1500 microscope with GFP filter. A ring of 150 mM Copper Sulphate, 2% SDS was use to prevent animals escaping from the plates. Unless indicated both Dar and Dar-defective animals were scored indifferently for the presence of SYTO13 labeling. The percentage of Dar animals was scored prior to SYTO13 labeling. To confirm that loss of SYTO13 labeling reflected a loss of *M. nematophilum* attachment wild type animals were washed from clearance assay plates after 90 minutes and restained with SYTO13. Approximately 10% of animals were SYTO13 positive indicating that *M. nematophilum* was no longer attached to the rectal opening of the majority these animals. Between 20 and 50 animals were scored per plate. Experiments were performed in triplicate and repeated at least three times. We observed some variability in the rate of pathogen clearance between experiments therefore assays were performed by two different people and data was only included if results were comparable. Furthermore, each graph only contains data from experiments where all genotypes presented were assayed in parallel.

### Microscopy

10–20 µl of settled, SYTO13 labeled animals were added to an equal volume of 600 mg/ml 2,3-Butanedione monoxime in M9 and mounted on 2% agarose pads. Adult animals expressing *tph-1p*::DSRED (*vsIs97*) were infected with virulent or avirulent forms of *M. nematophilum* and the first generation progeny were fixed using 4% paraformaldehyde and imaged by mounting on 2% agarose pads.

Animals were viewed on a Nikon Eclipse Ti inverted microscope using a Nikon ×40 objective (for SYTO13 labeled animals) or ×60 objective (for animals expressing *tph-1p*::DSRED). Images were obtained using Nikon NIS elements BR software. When *tph-1p*::DSRED expressing animals were used two images were acquired for each animal to allow the fluorescence intensity of ADF and NSM neuronal cell bodies to be measured.

Images were thresholded to highlight SYTO13 staining, or the ADF/NSM cell body, using Nikon NIS elements BR software and the average fluorescence intensity was the average pixel value within this thresholded area. Controls (wild type animals for SYTO13 experiments and uninfected animals for *tph-1p*::DSRED) were imaged in parallel and experiments were performed on at least three separate occasions. At least 30 animals were imaged per condition.

### Induction of heat shock-inducible transgenes

Expression from the heat shock promoter was achieved using two rounds of heat shock for 60 min separated by 30 min at 20°C. Heat shock was performed at 0, 24 and 48 hours after transfer of L1's to *M. nematophilum* plates when animals were at approximately L1, L2/3 and L3/4 stage respectively. A heat shock temperature of 33°C was used. Animals were allowed to recover at 20°C before scoring for the Dar phenotype when the animals reached adulthood.

### Statistical analysis

In all cases statistical analysis was performed using Prism 6 (GraphPad Software). The percentage of Dar animals was compared using an unpaired two-tailed t-test or one-way ANOVA followed by Tukey HSP Post hoc multiple comparison test. Food choice index data was compared using a one-way ANOVA followed by Tukey HSP Post hoc multiple comparison test. Clearance assay data was compared using a two-way ANOVA followed by Tukey HSP Post hoc multiple comparison test. * P≤0.05, ** P≤0.01, *** P≤0.001, **** P≤0.0001, n.s. P>0.05

## Supporting Information

Figure S1 **Expression of the rectal epithelial marker, LIN-48, is not altered by treatment with exogenous serotonin.** Animals carrying an integrated transgene expressing *lin-48p*::GFP were grown on control plates (A–C) or plates containing 3.8 mg/ml 5-HT (D–F) seeded with *E. Coli* OP50. Animals exposed to 5-HT for at least one generation were imaged. *lin-48p*::GFP expression was observed in phasmid sheath cells and K, K', F and U rectal epithelial cells. Expression of this transgene was not altered by treatment with 5-HT (compare B with E). The rectal opening is indicated with an arrow.
(TIF)

Figure S2 **TPH-1 is not required for behavioral avoidance of** ***M. nematophilum*** **.** The majority of wild type and *tph-1(n4622)* animals avoid bacterial lawns contaminated *M. nematophilum* under standard assay conditions (A). Animals were presented with a direct choice between *E. Coli* and *M. nematophilum* using a food choice assay (B). Using this assay wild type and *tph-1(n4622)* animals exhibit a strong preference for *E. Coli* after 4 hours (C).
(TIF)

Figure S3 **The Dar phenotype increases pathogen clearance rates but is not reversed by pathogen clearance.** A–C. Animals were mounted on 2% agarose pads immediately after being scored for the presence of SYTO13 labeled *M. nematophilum* at the 90 minute time point. No SYTO13 labeled *M. nematophilum* can be detected in 63% of wild type animals after 90 minutes on unseeded plates (Figure 1F and B and C) however these animals remain Dar (A and C). The rectal opening is indicated with an arrow. * indicates non-specific gut fluorescence. D. Wild type animals were infected with *M. nematophilum* on plates containing exogenous 5-HT and Dar and Dar-defective animals were separated prior to SYTO13 staining. The rate of clearance of SYTO13 labeled bacteria from the *C. elegans* rectal opening was measured. 5-HT treated Dar animals cleared the pathogen at a similar rate to wild type, untreated, control animals however the rate of pathogen clearance was significantly decreased in 5-HT treated Dar-defective animals.
(TIF)

Figure S4 **Increased expression of TPH-1 in NSM neurosecretory neurons is caused by reduced contact with contaminated bacterial lawns.** Wild type and *egl-30(ad805)* animals carrying an integrated *tph-1p*::DSRED transgene were infected with *M. nematophilum* or an avirulent form of *M. nematophilum* using standard (small lawn) or “big lawn” assay conditions. The mean *tph-1p*::DSRED fluorescence in NSM neurons was quantified. Expression of *tph-1p*::DSRED was significantly increased when wild type animals were grown on plates contaminated with virulent *M. nematophilum*. This increase in expression was not observed under conditions when animals were unable to leave the bacterial lawn, in *egl-30(ad805)* animals or when wild type animals were infected on “big lawns”.
(TIF)

Figure S5 **Two serotonin receptors, SER-1 and SER-7, act redundantly to suppress the Dar phenotype.** Adult animals of the indicated genotypes were exposed to *M. nematophilum* on plates containing exogenous serotonin and the Dar phenotype was scored in their progeny. Serotonin treatment of wild type, *ser-1(ok345)*, *ser-4(ok512)*, *ser-7(ok1944)*, *ser-7(tm1325)* and *mod-1(ok103)* mutants infected with *M. nematophilum* caused a significant decrease in the percentage of Dar animals (A). The effect of exogenous serotonin was suppressed in *ser-1(ok345);ser-7(tm1325)* double mutants (A). When animals were infected on lawns contaminated with 0.05% *M. nematophilum* the Dar phenotype was increased in *ser-1(ok345);ser-7(tm1325)* double mutants when compared to wild type controls (B). The Dar phenotype was not significantly altered in any single mutants tested (B). *ser-1(ok345);ser-7(tm1325)* animals cleared SYTO13 labeled pathogen at a similar rate to wild type animals. Unlike wild type animals, this clearance rate was not altered by treatment with exogenous 5-HT (C).
(TIF)

Table S1 **Dar phenotype of strains that phenocopy the effect of exogenous serotonin.**
(DOCX)

Table S2 ***C. elegans*** **strains used in this study.**
(DOCX)
