# Supplementary figures and images for: Serotonergic Chemosensory Neurons Modify the C. elegans Immune Response by Regulating G-Protein Signaling in Epithelial Cells
Source: PLoS Pathog. 2013 Dec 12;9(12):e1003787. doi: 10.1371/journal.ppat.1003787 (PMC3861540; doi:10.1371/journal.ppat.1003787)

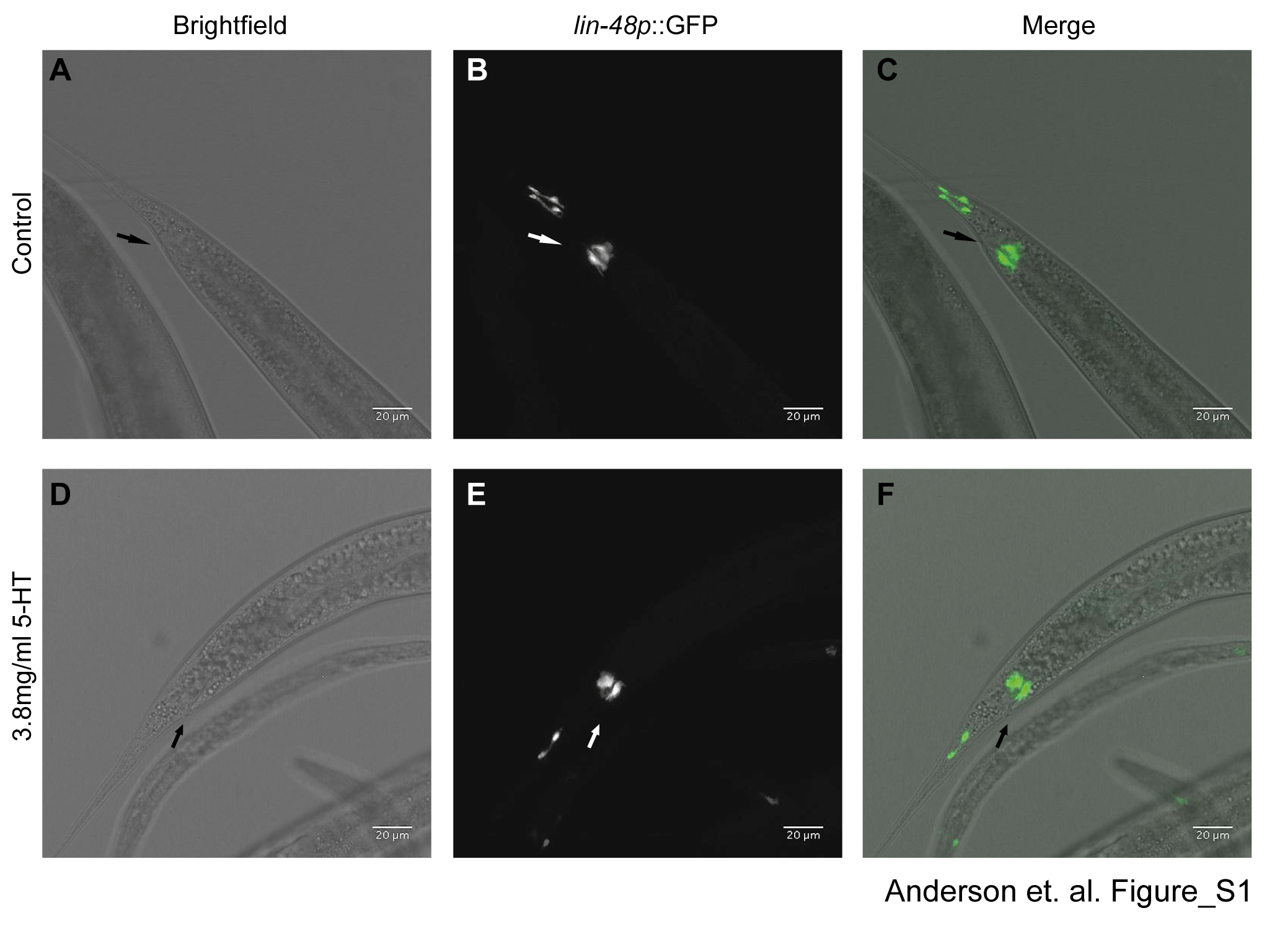

Supplement: Figure S1 — Expression of the rectal epithelial marker, LIN-48, is not altered by treatment with exogenous serotonin. Animals carrying an integrated transgene expressing lin-48p::GFP were grown on control plates (A–C) or plates containing 3.8 mg/ml 5-HT (D–F) seeded with E. Coli OP50. Animals exposed to 5-HT for at least one generation were imaged. lin-48p::GFP expression was observed in phasmid sheath cells and K, K', F and U rectal epithelial cells. Expression of this transgene was not altered by treatment with 5-HT (compare B with E). The rectal opening is indicated with an arrow. (TIF) [file ppat.1003787.s001.tif]

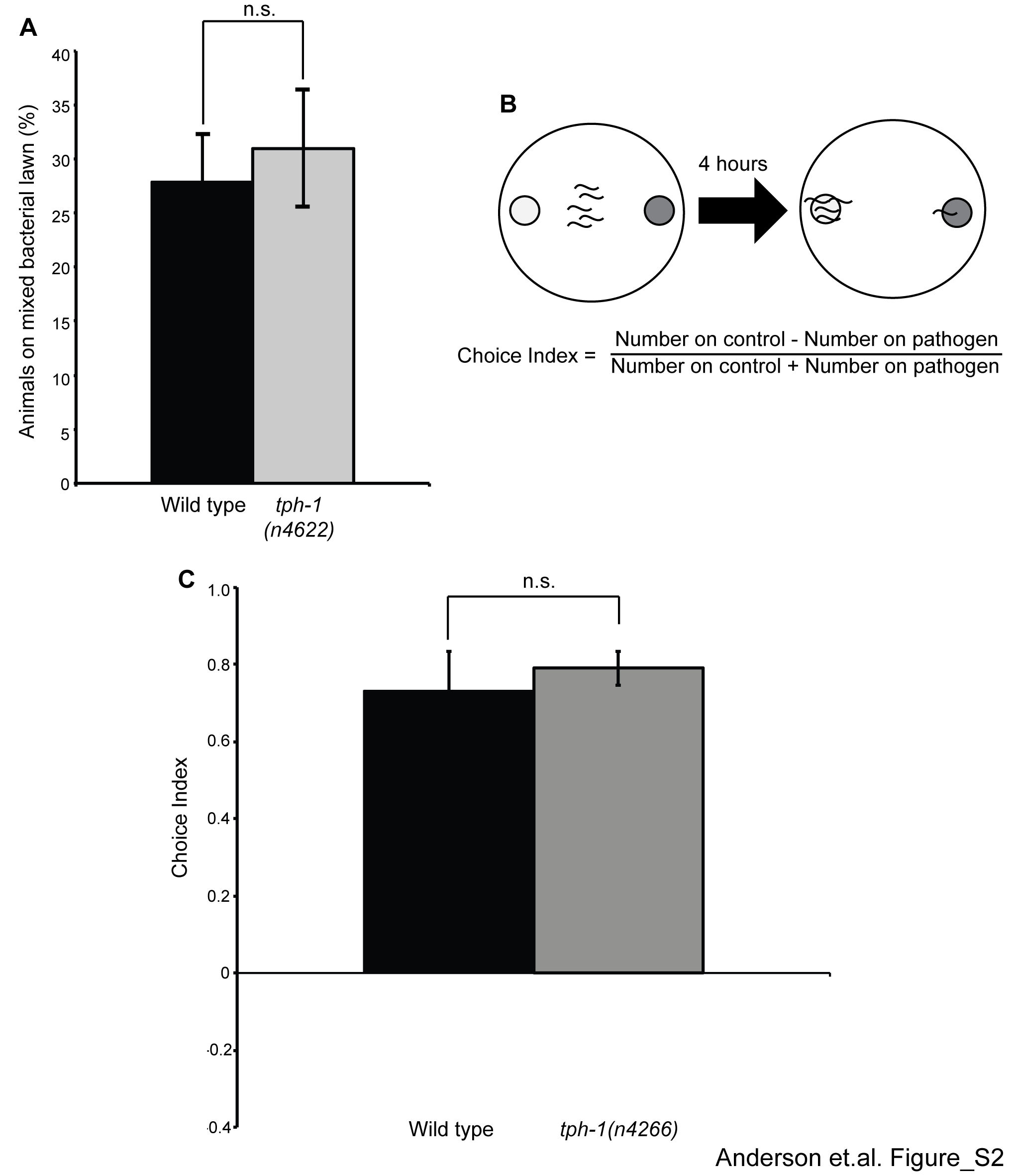

Supplement: Figure S2 — TPH-1 is not required for behavioral avoidance of M. nematophilum . The majority of wild type and tph-1(n4622) animals avoid bacterial lawns contaminated M. nematophilum under standard assay conditions (A). Animals were presented with a direct choice between E. Coli and M. nematophilum using a food choice assay (B). Using this assay wild type and tph-1(n4622) animals exhibit a strong preference for E. Coli after 4 hours (C). (TIF) [file ppat.1003787.s002.tif]

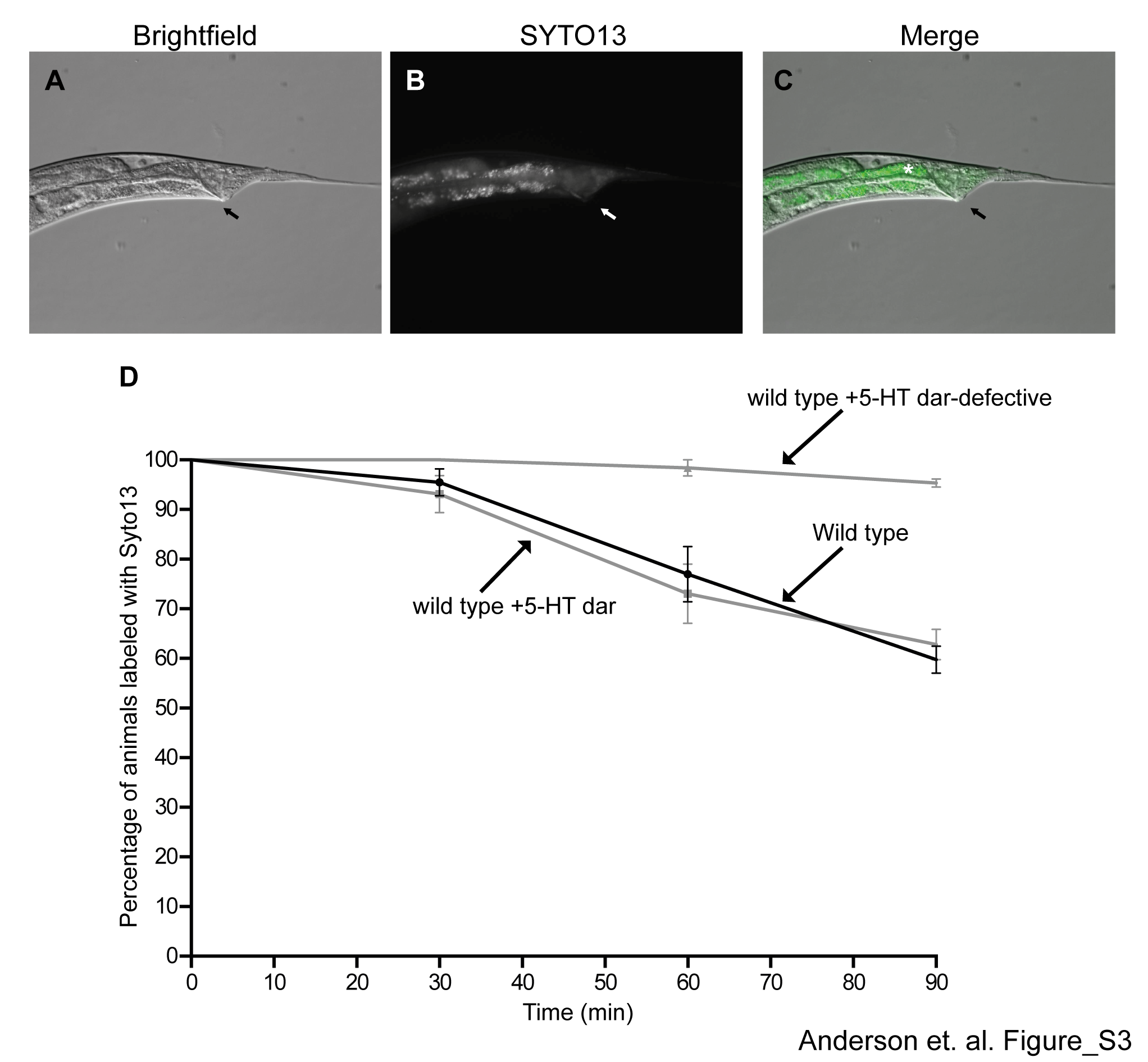

Supplement: Figure S3 — The Dar phenotype increases pathogen clearance rates but is not reversed by pathogen clearance. A–C. Animals were mounted on 2% agarose pads immediately after being scored for the presence of SYTO13 labeled M. nematophilum at the 90 minute time point. No SYTO13 labeled M. nematophilum can be detected in 63% of wild type animals after 90 minutes on unseeded plates (Figure 1F and B and C) however these animals remain Dar (A and C). The rectal opening is indicated with an arrow. * indicates non-specific gut fluorescence. D. Wild type animals were infected with M. nematophilum on plates containing exogenous 5-HT and Dar and Dar-defective animals were separated prior to SYTO13 staining. The rate of clearance of SYTO13 labeled bacteria from the C. elegans rectal opening was measured. 5-HT treated Dar animals cleared the pathogen at a similar rate to wild type, untreated, control animals however the rate of pathogen clearance was significantly decreased in 5-HT treated Dar-defective animals. (TIF) [file ppat.1003787.s003.tif]

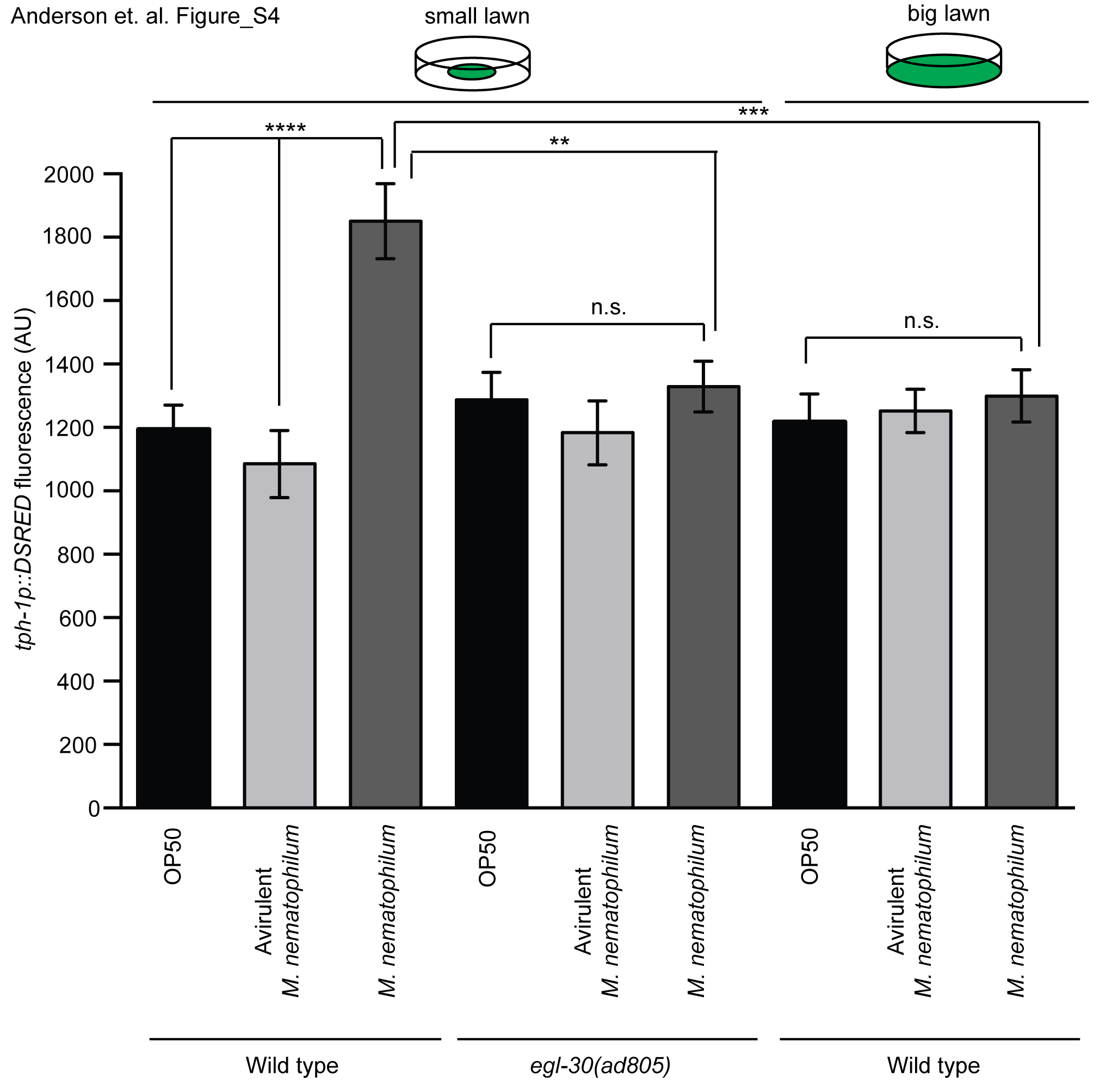

Supplement: Figure S4 — Increased expression of TPH-1 in NSM neurosecretory neurons is caused by reduced contact with contaminated bacterial lawns. Wild type and egl-30(ad805) animals carrying an integrated tph-1p::DSRED transgene were infected with M. nematophilum or an avirulent form of M. nematophilum using standard (small lawn) or “big lawn” assay conditions. The mean tph-1p::DSRED fluorescence in NSM neurons was quantified. Expression of tph-1p::DSRED was significantly increased when wild type animals were grown on plates contaminated with virulent M. nematophilum. This increase in expression was not observed under conditions when animals were unable to leave the bacterial lawn, in egl-30(ad805) animals or when wild type animals were infected on “big lawns”. (TIF) [file ppat.1003787.s004.tif]

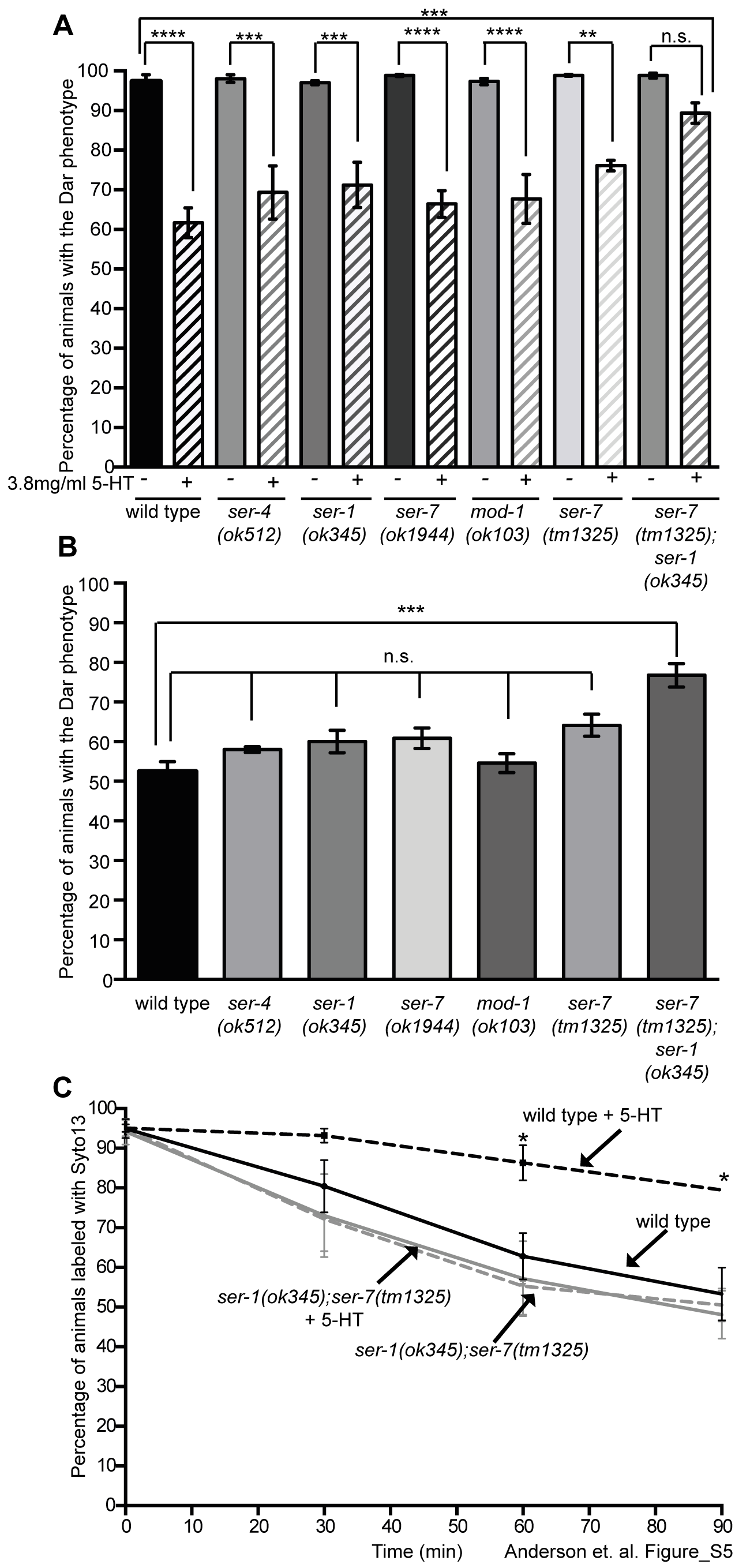

Supplement: Figure S5 — Two serotonin receptors, SER-1 and SER-7, act redundantly to suppress the Dar phenotype. Adult animals of the indicated genotypes were exposed to M. nematophilum on plates containing exogenous serotonin and the Dar phenotype was scored in their progeny. Serotonin treatment of wild type, ser-1(ok345), ser-4(ok512), ser-7(ok1944), ser-7(tm1325) and mod-1(ok103) mutants infected with M. nematophilum caused a significant decrease in the percentage of Dar animals (A). The effect of exogenous serotonin was suppressed in ser-1(ok345);ser-7(tm1325) double mutants (A). When animals were infected on lawns contaminated with 0.05% M. nematophilum the Dar phenotype was increased in ser-1(ok345);ser-7(tm1325) double mutants when compared to wild type controls (B). The Dar phenotype was not significantly altered in any single mutants tested (B). ser-1(ok345);ser-7(tm1325) animals cleared SYTO13 labeled pathogen at a similar rate to wild type animals. Unlike wild type animals, this clearance rate was not altered by treatment with exogenous 5-HT (C). (TIF) [file ppat.1003787.s005.tif]
