# Supplementary material for: Serotonergic Chemosensory Neurons Modify the C. elegans Immune Response by Regulating G-Protein Signaling in Epithelial Cells
Source: PLoS Pathog. 2013 Dec 12;9(12):e1003787. doi: 10.1371/journal.ppat.1003787 (PMC3861540; doi:10.1371/journal.ppat.1003787)
Supplement: Table S1 — Dar phenotype of strains that phenocopy the effect of exogenous serotonin. (DOCX) [file ppat.1003787.s006.docx]

| **Strain** | **Relevant Genotype** | **Relevant Phenotype** | **Percentage Dar (± s.e.m.)** |
| --- | --- | --- | --- |
| N2 | Wild Type |  | 94.40 ± 1.46 |
| CB270 | *unc-42(e270)* | *egl-c, unc* | 98.41 ± 1.03 |
| CB113 | *unc-17(e113)* | *egl-c, unc* | 94.49 ± 1.36 |
| CB189 | *unc-32(e189)* | *egl-c, unc* | 98.57 ± 1.09 |
| DR96 | *unc-76(e911)* | *egl-c, unc* | 100 ± 0 |
| DA695 | *egl-19(ad695)* | *egl-c, unc* | 100 ± 0 |
| DR1089 | *unc-77(e625)* | *egl-c, unc* | 100 ± 0 |
| MT7929 | *unc-13(e51)* | *unc* | 94.55 ± 4.55 |

**Table S1 Dar phenotype of strains that phenocopy the effect of exogenous serotonin**

The number of Dar animals was scored as a percentage of the total. Values are means +/- the standard error.
