# Supplementary material for: Serotonergic Chemosensory Neurons Modify the C. elegans Immune Response by Regulating G-Protein Signaling in Epithelial Cells
Source: PLoS Pathog. 2013 Dec 12;9(12):e1003787. doi: 10.1371/journal.ppat.1003787 (PMC3861540; doi:10.1371/journal.ppat.1003787)
Supplement: Table S2 — C. elegans strains used in this study. (DOCX) [file ppat.1003787.s007.docx]

**Table S2 *C. elegans* strains used in this study**

| **Strain** | **Relevant Genotype** | **Source** | **Reference** |
| --- | --- | --- | --- |
| N2 Bristol | Wild Type | CGC | Brenner 1974 [1] |
| GR1321 | *tph-1(mg280)* | CGC | Sze 2000 [2] |
| QL101 | *tph-1(n4622)* | QueeLim Chung  (Kings College London) | Shivers 2009 [3] |
| CX6732 | *tph-1(mg280);kyEx945*(ceh-2p∷tph-1∷GFP) | Cornelia Bargmann  (HHMI, Rockerfeller University) | Zhang 2005 [4] |
| CX6736 | *tph-1(mg280);kyEx948*(srh-142p∷tph-1∷GFP) | Cornelia Bargmann  (HHMI, Rockerfeller University) | Zhang 2005 [4] |
| CX7572 | *tph-1(mg280);kyEx999*(srh-142p∷tph-1∷GFP, ceh-2p∷tph-1∷GFP) | Cornelia Bargmann  (HHMI, Rockerfeller University) | Zhang 2005 [4] |
| RJM21 | *impEx004*(egl-5p∷EGL-30*) | Stephen Nurrish (University College London) | McMullan 2012 [5] |
| LX960 | *lin-15B(n765);vsIs97* (tph-1p∷DSRed,lin-15(+)) | CGC | Tanis 2008 (supplement) [6] |
| DA823 | *egl-30(ad805)* | CGC | Bastiani 2003 [7] |
| MT8504 | *egl-10(md176)* | CGC | Koelle and Horvitz 1996 [8] |
| MT1443 | *egl-10(n692)* | CGC | Koelle and Horvitz 1996 [8] |
| QT956 | *nzEx483*(egl-5p∷RHO-1*) | Stephen Nurrish (University College London) | McMullan 2012 [5] |
| CB6603 | *eIs102*(egl-5p∷LIN-45*) | Jonathan Hodgkin (University of Oxford) |  |
| CM117 | *saIs14 (*lin-48p∷GFP) | Sophie Jarriault (IGBMC, Strasbourg) | Johnson 2001 [9] |
| DA1814 | *ser-1(ok345)* | CGC | Xiao 2006 [10] |
| AQ866 | *ser-4(ok512)* | CGC | Carre-Pierrat 2006 [11] |
| DA2100 | *ser-7(tm1325)* | CGC | Hobson 2006 [12] |
| RB1585 | *ser-7(ok1944)* | CGC | Li et. al. 2012 [13] |
| DA2109 | *ser-1(ok345);ser-7(tm1325)* | CGC | Hobson 2006 [12] |
| MT9668 | *mod-1(ok103)* | CGC | Ranganathan 2000 [14] |
| CB270 | *unc-42(e270)* | CGC | Brenner 1974 [1] |
| CB113 | *unc-17(e113)* | CGC | Brenner 1974 [1] |
| CB189 | *unc-32(e189)* | CGC | Brenner 1974 [1] |
| DR96 | *unc-76(e911)* | CGC | Desai 1988 [15] |
| DA695 | *egl-19(ad695)* | CGC | Kwok 2008 [16] |
| DR1089 | *unc-77(e625)* | CGC | Yeh 2008 [17] |
| MT7929 | *unc-13(e51)* | CGC | Brenner 1974 [1] |

**Supplemental References**

1. Brenner S (1974) The genetics of *Caenorhabditis elegans*. Genetics 77: 71–94.

2. Sze J, Victor M, Loer C, Shi Y, Ruvkun G (2000) Food and metabolic signalling defects in a *Caenorhabditis elegans* serotonin-synthesis mutant. Nature 403: 560–564.

3. Shivers R, Kooistra T, Chu S, Pagano D, Kim D (2009) Tissue-specific activities of an immune signaling module regulate physiological responses to pathogenic and nutritional bacteria in *C. elegans*. Cell Host Microbe 6: 321–351.

4. Zhang Y, Lu H, Bargmann CI (2005) Pathogenic bacteria induce aversive olfactory learning in *Caenorhabditis elegans*. Nature 438: 179–184. doi:10.1038/nature04216.

5. McMullan R, Anderson A, Nurrish S (2012) Behavioral and Immune Responses to Infection Require Gαq- RhoA Signaling in *C. elegans*. PLoS Pathog 8: e1002530. doi:10.1371/journal.ppat.1002530.

6. Tanis JEJ, Moresco JJJ, Lindquist RAR, Koelle MRM (2008) Regulation of serotonin biosynthesis by the G proteins Galphao and Galphaq controls serotonin signaling in *Caenorhabditis elegans*. Genetics 178: 157–169. doi:10.1534/genetics.107.079780.

7. Bastiani CAC, Gharib SS, Simon MIM, Sternberg PWP (2003) *Caenorhabditis elegans* Galphaq regulates egg-laying behavior via a PLCbeta-independent and serotonin-dependent signaling pathway and likely functions both in the nervous system and in muscle. Genetics 165: 1805–1822.

8. Koelle MR, Horvitz HR (1996) EGL-10 regulates G protein signaling in the *C. elegans* nervous system and shares a conserved domain with many mammalian proteins. Cell 84: 115–125. doi:10.1016/S0092-8674(00)80998-8.

9. Johnson AD, Fitzsimmons D, Hagman J, Chamberlin HM (2001) EGL-38 Pax regulates the ovo-related gene lin-48 during *Caenorhabditis elegans* organ development. Development 128: 2857–2865.

10. Xiao HH, Hapiak VMV, Smith KAK, Lin LL, Hobson RJR, et al. (2006) SER-1, a *Caenorhabditis elegans* 5-HT"2-like receptor, and a multi-PDZ domain containing protein (MPZ-1) interact in vulval muscle to facilitate serotonin-stimulated egg-laying. Dev Biol 298: 13–13. doi:10.1016/j.ydbio.2006.06.044.

11. Carre-Pierrat M, Baillie D, Johnsen R, Hyde R, Hart A, et al. (2006) Characterization of the *Caenorhabditis elegans* G protein-coupled serotonin receptors. Invert Neurosci 6: 189–205. doi:10.1007/s10158-006-0033-z.

12. Hobson RJR, Hapiak VMV, Xiao HH, Buehrer KLK, Komuniecki PRP, et al. (2006) SER-7, a *Caenorhabditis elegans* 5-HT7-like receptor, is essential for the 5-HT stimulation of pharyngeal pumping and egg laying. Genetics 172: 159–169. doi:10.1534/genetics.105.044495.

13. Li Z, Li Y, Yi Y, Huang W, Yang S, et al. (2012) Dissecting a central flip-flop circuit that integrates contradictory sensory cues in *C. elegans* feeding regulation. Nature Communications 3: 776.

14. Ranganathan R, Cannon SC, Horvitz HR (2000) MOD-1 is a serotonin-gated chloride channel that modulates locomotory behaviour in *C. elegans*. Nature 408: 470–475. doi:10.1038/35044083.

15. Desai C, Garriga G, McIntire SL, Horvitz HR (1988) A genetic pathway for the development of the *Caenorhabditis elegans* HSN motor neurons. Nature 336: 638–646. doi:10.1038/336638a0.

16. Kwok TCY, Hui K, Kostelecki W, Ricker N, Selman G, et al. (2008) A genetic screen for dihydropyridine (DHP)-resistant worms reveals new residues required for DHP-blockage of mammalian calcium channels. PLoS Genet 4: e1000067–e1000067. doi:10.1371/journal.pgen.1000067.

17. Yeh E, Ng S, Zhang M, Bouhours M, Wang Y, et al. (2008) A putative cation channel, NCA-1, and a novel protein, UNC-80, transmit neuronal activity in *C. elegans*. PLoS Biol 6: e55. doi:10.1371/journal.pbio.0060055.
